# Effective microwave-assisted approach to 1,2,3-triazolobenzodiazepinones via tandem Ugi reaction/catalyst-free intramolecular azide–alkyne cycloaddition

**DOI:** 10.3762/bjoc.17.57

**Published:** 2021-03-08

**Authors:** Maryna O Mazur, Oleksii S Zhelavskyi, Eugene M Zviagin, Svitlana V Shishkina, Vladimir I Musatov, Maksim A Kolosov, Elena H Shvets, Anna Yu Andryushchenko, Valentyn A Chebanov

**Affiliations:** 1 Division of Chemistry of Functional Materials, State Scientific Institution “Institute for Single Crystals” of National Academy of Sciences of Ukraine, 60 Nauky Ave, Kharkiv, 61072, Ukraine; 2 Department of Chemistry, V. N. Karazin Kharkiv National University, 4 Svobody Sq., Kharkiv, 61022, Ukraine; 3 Department of Chemistry, University of Michigan – Ann Arbor, 930 North University Ave, Ann Arbor, MI 48109, USA; 4 Department of Chemistry, University of Nebraska – Lincoln, 639 N 12th St, Lincoln, NE 68588, USA

**Keywords:** click chemistry, microwave chemistry, multicomponent reactions, triazolobenzodiazepines, Ugi reaction

## Abstract

A novel catalyst-free synthetic approach to 1,2,3-triazolobenzodiazepinones has been developed and optimized. The Ugi reaction of 2-azidobenzaldehyde, various amines, isocyanides, and acids followed by microwave-assisted intramolecular azide–alkyne cycloaddition (IAAC) gave a series of target heterocyclic compounds in moderate to excellent yields. Surprisingly, the normally required ruthenium-based catalysts were found to not affect the IAAC, only making isolation of the target compounds harder while the microwave-assisted catalyst-free conditions were effective for both terminal and non-terminal alkynes.

## Introduction

**Figure 1:**
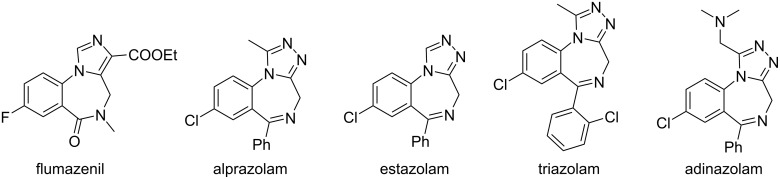
Benzodiazepine-based azolo-containing drugs.

Benzannulated heterocycles are among the most important molecular frameworks in medicinal chemistry. Of these, 1,4-benzodiazepines fused with pyrazole or triazole cycles are the base for many drugs (Figure 1) [1]. The spectrum of their biological activity includes tranquilizing, muscular relaxant, anticonvulsant, and sedative effects [2]. The general application of 1,2,4-triazolobenzodiazepines is the treatment of central nervous system (CNS) disorders. Such drugs as alprazolam and estazolam are used as anxiolytic agents, whereas adinazolam is known as an antidepressant [3]. Benzodiazepine molecules are ligands for GABA-receptors and act as positive allosteric modulators to enhance the effect of the neurotransmitter. That results in sedative, hypnotic (sleep-inducing), anxiolytic (anti-anxiety), anticonvulsant, and muscle relaxant effects of these drugs [4].

Benzodiazepines and their derivatives are also known to be antagonists for the cholecystokinin-1 receptor (CCK1R) located in the gastrointestinal tract. It is a potential target for treating obesity and diabetes [5].

**Figure 2:**
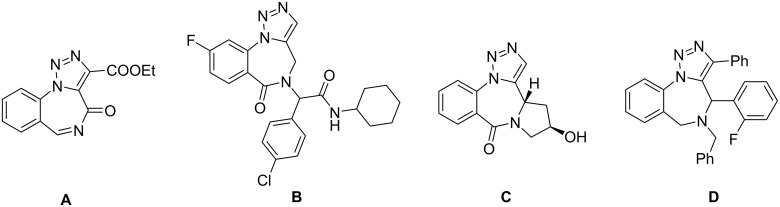
Novel potential 1,2,3-triazolobenziadiazepine drugs.

Although derivatives of 1,2,3-triazolobenzodiazepines are less studied as to their 1,2,4-triazole fused analogs, 1,2,3-triazolobenzodiazepinone **A** (Figure 2) has already reached clinical success in the treatment of CNS disorders [6]. Preliminary studies of the biological activity of other diversely substituted 1,2,3-triazolobenzodiazepines demonstrate their similar properties with all main categories of 1,2,4-triazolobenzodiazepine-based drugs [7]. For example, compound **B** demonstrated high anticonvulsant activity after tests in silico and in vivо [7]. Moreover, compounds **C** and **D** reveal high activity as casein kinase 2 (CK2) inhibitor and high antitumor activity which makes compounds to be promising anticancer drugs [8].

There are quite a few methods for the synthesis of the benzodiazepine skeleton [9]. But these days more and more research groups significantly shift their efforts towards the wide application of a tandem synthetic approach to solve different matters. This approach consists of a combination of several versatile reactions allowing assembling complex molecules just in a few steps and constitutes one of the most actual and promising synthetic routes of modern organic chemistry.

**Scheme 1:**
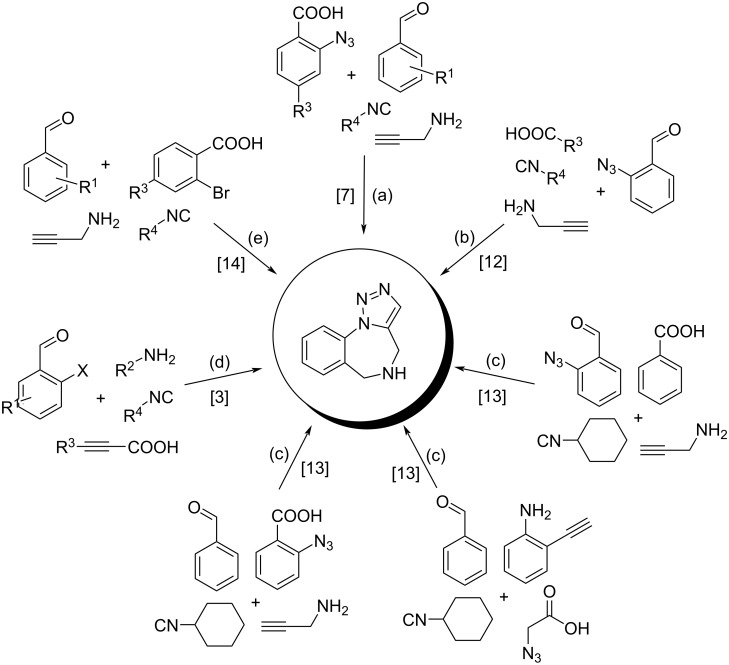
Examples of synthesis of 1,2,3-triazolobenzodiazepines via tandem approach Ugi reaction/IAAC. Reagents and conditions: (a) MeOH, reflux, 24 h; (b) (1) 4 Å MS, EtOH, rt, 20 h, (2) Base, rt, 30 min, (3) EtOH, reflux, 3 h; (c) (1) MeOH, 24–48 h, rt, (2) PhH, reflux, 4–18 h; (d) (1) MeOH, rt, 8 h; (2) NaN_3_, CuI, ʟ-proline, DMSO, 120 °C, 24 h; (e) (1) MeOH, rt, 24 h, (2) NaN_3_, CuI, DMSO, 100 °C, 3 h.

Among a wide variety of tandem strategies combination of an isocyanide-based multicomponent Ugi reaction with secondary transformations is one of the most powerful tools and provides access to a large number of diverse heterocyclic compounds [10–11]. Over the past decade, several cases of using an Ugi four-component reaction (Ugi-4CR) in combination with intramolecular azide–alkyne cycloaddition (IAAC) for the synthesis of 1,2,3-triazolobenzodiazepines were reported [3,7,12–14] (Scheme 1).

Even though at first glance the syntheses from Scheme 1, where in the second step the NaN_3_ is used (Scheme 1, (d) and (e)) [3,14], are the combination of sequential Ugi and IAAC reactions, that’s not entirely true. Actually, the mechanism of the abovementioned Cu-catalyzed reactions with NaN_3_ includes a [3 + 2] click reaction between the azide ion with the triple bond and further C–N coupling instead of the IAAC reaction.

Compounds having no fused benzene ring or with a heterocyclic moiety instead could also be obtained via the tandem approach Ugi reaction/IAAC [15–16].

Despite the availability of previously described methods for the synthesis of 1,2,3-triazolobenzodiazepines represented in Scheme 1, they have such drawbacks as long reaction time, use of toxic solvents, additional catalysts, etc.

In this article, we present a novel tandem Ugi/catalyst-free intramolecular azide–alkyne cycloaddition approach to the synthesis of 1,2,3-triazolobenzodiazepinones.

## Results and Discussion

The development of the tandem Ugi/Click reaction approach for 1,2,3-triazolobenzodiazepinone synthesis can be logically divided into two principal parts: modification and performing synthesis of Ugi-reaction products from substrates with preinstalled functionality for IAAC (azide and alkyne groups) and then optimizing the conditions for the azide–alkyne cycloaddition that should lead to the desired compounds.

**Scheme 2:**
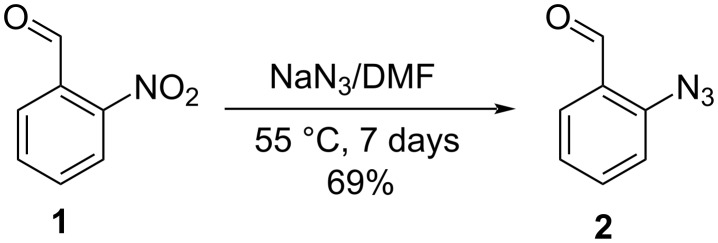
Azide precursor synthesis.

The key precursor in our study bearing an azide functional group is 2-azidobenzaldehyde **2,** which can be prepared via two similar procedures previously described in the literature [17–18]. Our synthetic route is based on the protocol offered in T. Pelkey’s publication [18] using DMF as a solvent for the nucleophilic aromatic substitution of the nitro group in 2-nitrobenzaldehyde (**1**) instead of the more efficient but also more toxic HMPA. A few modifications made in the original procedure in combination with column chromatography led to pure product **2** in good yield (Scheme 2).

**Scheme 3:**
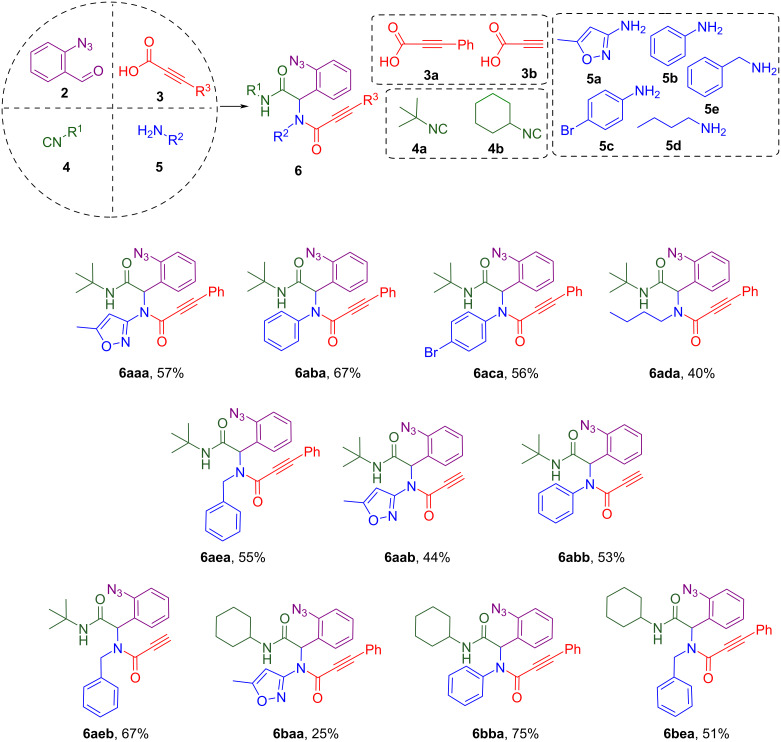
Synthesis of Ugi products **6**, their structures and yields.

With azide precursor **2** in hand as an aldehyde component, the Ugi reaction was performed with various isocyanides **4**, amines **5**, and phenylpropargylic acid (**3a**) or propargylic acid (**3b**, Scheme 3).

In most cases, the reactions were carried under standard conditions for Ugi-4CR mentioned in the literature. Aldehyde and amine were premixed for 2 hours in methanol, then acid and isocyanide were sequentially added. Stirring the reaction mixture for 24 hours at room temperature led to the formation of Ugi products **6** that usually didn’t require any purification. But trying to perform the abovementioned procedure with aliphatic amines **5d,e**, we faced difficulties that the desired product didn’t precipitate from the reaction mixture. Therefore, several alternative procedures were tested, and refluxing the mixture of the starting materials in DCM in an oil bath for 2–3 days with further purification gave the best results. Gratifyingly, in most cases *N*-(1-(2-azidophenyl)propiolamides **6** were obtained in moderate yields (Scheme 3).

**Figure 3:**
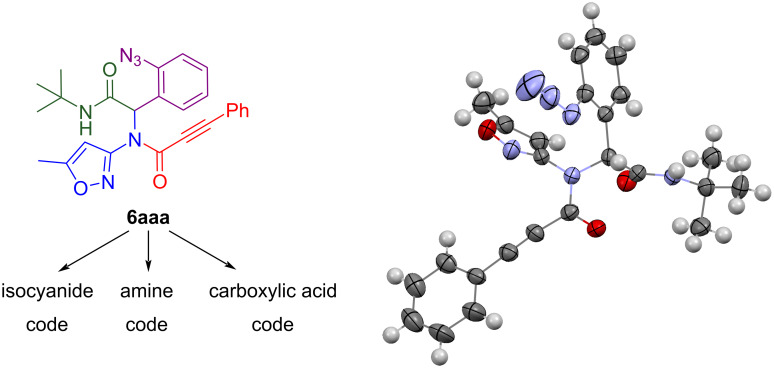
Code legend for Ugi products **6** and molecular structure (X-ray analysis) of compound **6aaa**.

Ugi product **6aaa** (first letter – isocyanide code, second letter – amine code, third – carboxylic acid code) was the first to isolate in our trials and its structure was confirmed by X-ray analysis (Figure 3) in addition to mass spectrometry, elemental analysis, ^1^H and ^13^C NMR data. Compound **6** containing an isoxazole moiety is of key interest in this study since previously our group reported [19] a successful usage of 3-aminoizoxazoles as unusual amine components in the Ugi-4CR reaction and here we continue to build molecular diversity of isoxazole-derived Ugi products and their successors. Therefore, namely compound **6aaa** was chosen as a model substrate for the conditions screening.

It is also worth mentioning that the structure of Ugi product **6aea** obtained by an alternative method in an oil bath in DCM was also confirmed by X-ray analysis (see Supporting Information File 1).

**Scheme 4:**
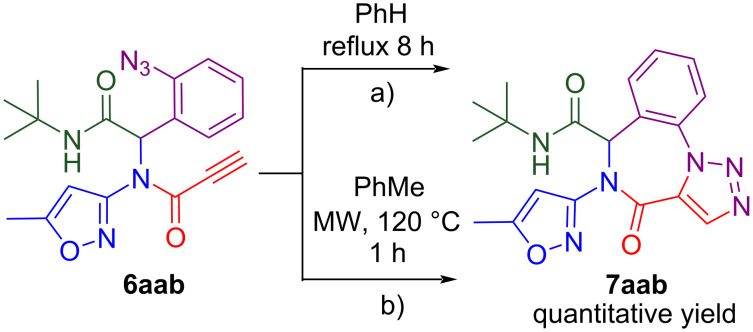
Cyclization of Ugi-product **6aab** with terminal alkyne fragment.

In the next step of our study, the Ugi products **6** were involved in IAAC. Compounds such as **6aab**, **6abb** and **6aeb** with terminal alkyne fragment can be easily cyclized under thermal uncatalyzed conditions in various solvents – from nonpolar benzene to polar protic solvents, and even in water, depending on substrate solubility [20]. Firstly, we used a procedure similar to described by I. Akritopoulou-Zanze et al. [13] for cyclization: compound **6aab** was refluxed in benzene for 8 hours until TLC monitoring demonstrated the full transformation of starting material into a new compound. The evaporation of the solution gave a white powder of compound **7aab** in quantitative yield (Scheme 4a).

**Figure 4:**
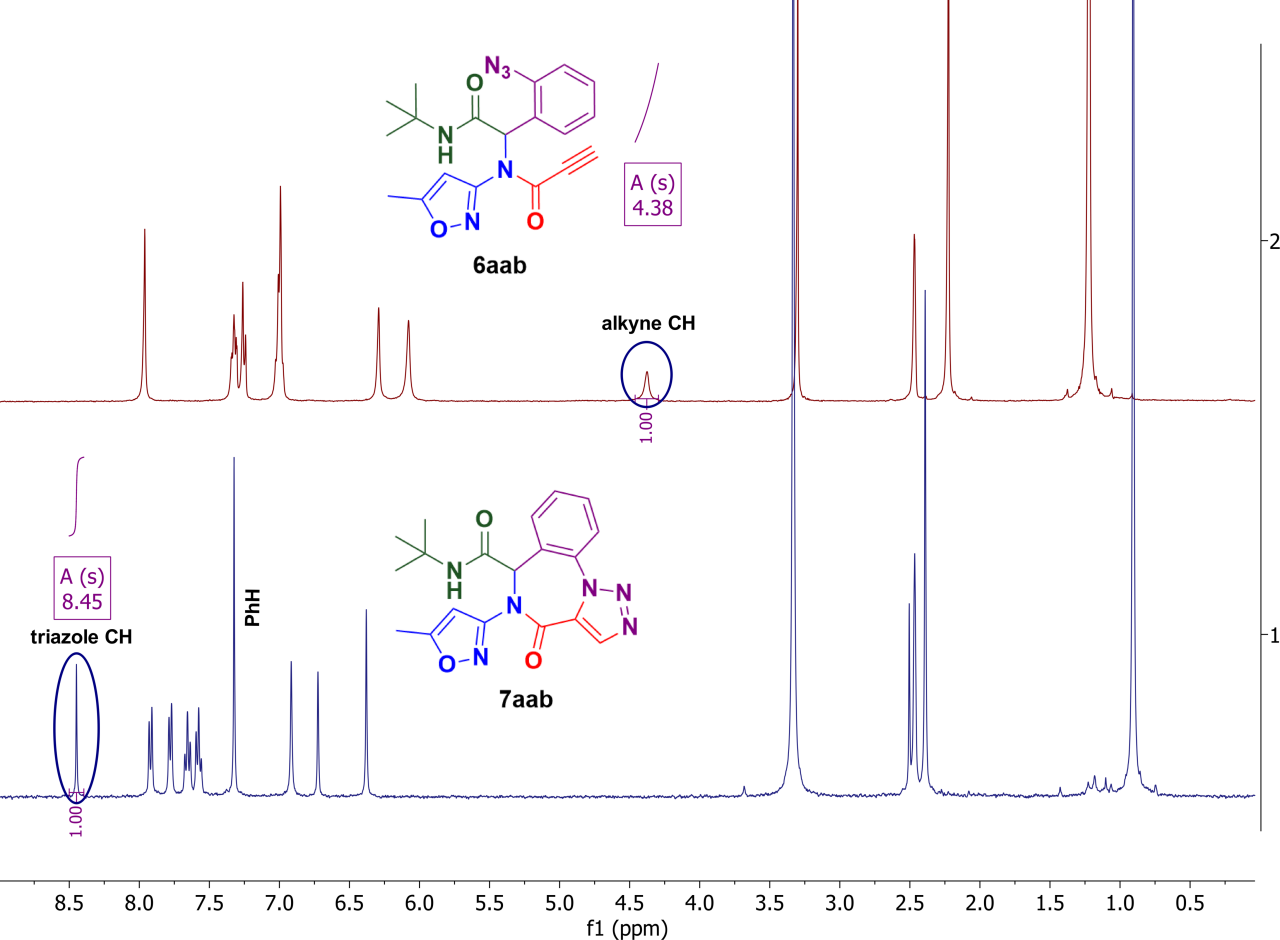
^1^H NMR spectra of the reactant and the product of IAAC.

The formation of compound **7aab** can be easily proved by comparing its ^1^H NMR spectrum with the spectrum of the initial substrate **6aab** (Figure 4). There is clear evidence of alkyne proton signal (4.38 ppm) disappearance and a new proton signal rising at 8.45 ppm (chemical shift is close to triazole proton value), that can indicate the transformation of the alkyne moiety to the triazole ring. The signals of the aromatic ring also significantly change: the aromatic multiplets observed in the ^1^H NMR spectrum of the starting material turn into two well-resolved doublets and two triplets slightly shifted downfield. This phenomenon can be explained by the loss of conformational motion of the aromatic ring and the formation of the hard molecular carcass of the 7-membered ring fused with a 1,2,3-triazole cycle.

**Scheme 5:**
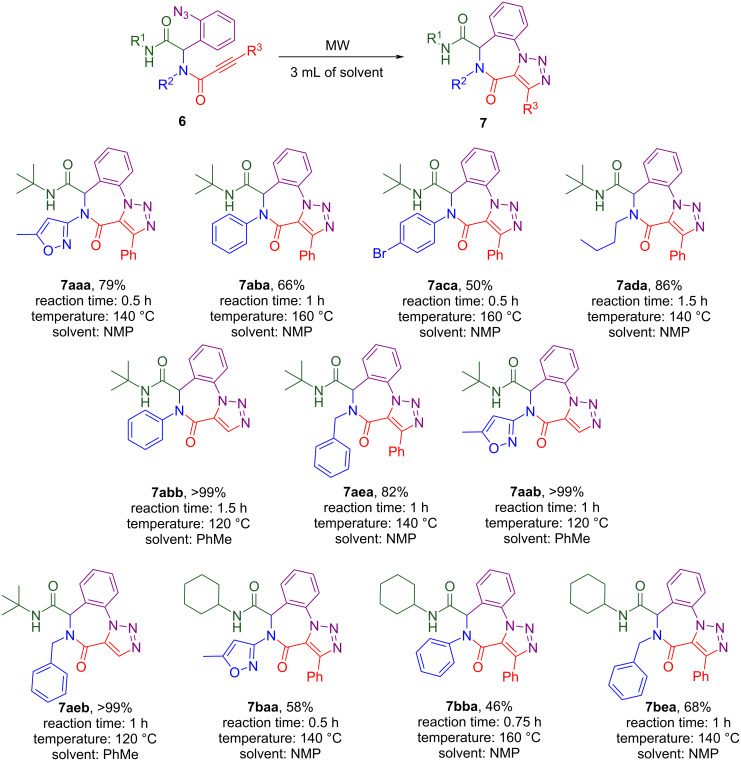
The substrate scope of intermolecular cycloaddition.

Then we applied higher temperature conditions for the cyclization, performing a reaction under microwave irradiation in a sealed vial, and changed the solvent to less toxic (than benzene) toluene. That led to a decreasing reaction time to 1–1.5 hours (the reaction progress was monitored by TLC) with no impact on the product purity and yield. Quantitative yields were also observed when *N*-phenyl (**6abb**) and *N*-benzyl (**6aeb**) propiolamides were subjected to the cyclization (see results on Scheme 5).

Performing the IAAC for internal alkynes under non-catalyzed conditions is more challenging due to the reduced reactivity of the triple bond. Usually, AAC reactions on non-terminal alkynes are performed with ruthenium catalysis [21] that determined our decision to start screening conditions using the chloro(cyclopentadienyl)bis(triphenylphosphine)ruthenium(II) complex ((Cp)Ru(PPh_3_)_2_Cl) as catalyst. However, carrying out the reaction with the catalyst under different conditions (variation of solvent and temperature) failed to yield the desired cyclized product from **6aaa** (Table 1, part A). Reactions at room temperature and higher produced an only inseparable and unidentifiable mixture of side products, and at low temperatures (Table 1, entries 2 and 3) no transformation of the starting material was observed. Only at higher temperatures (Table 1, entries 6 and 7) possibly traces of the product were fixed (new TLC spot) but still inseparable and not observed in ^1^H NMR.

**Figure 5:**
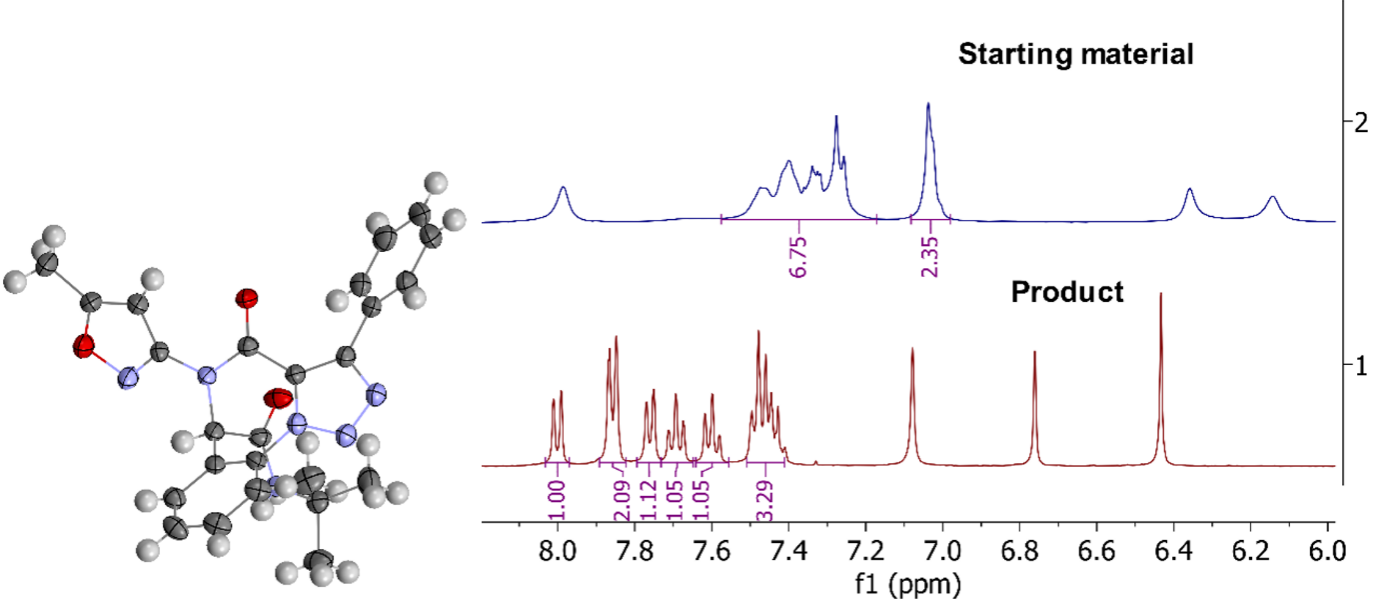
Molecular structure of compound **7aaa** (X-ray analysis) and comparison of ^1^H NMR spectra of compounds **6aaa** and **7aaa**.

Unexpectedly, refluxing compound **6aaa** in benzene for 12 hours without any catalyst (Table 1, part B, entry 8) produced a white precipitate that, according to X-ray analysis, was the target triazolobenzodiazepinone **7aaa** (Figure 5). Similarly, the IAAC of the Passerini product under catalyst-free conditions was described earlier [22]. The ^1^H NMR analysis is less descriptive than in the case with IAAC on terminal alkyne due to the lack of transformation of the alkyne proton into a triazole one that was tracked for compounds **7aab**, **7abb** and **7aeb** previously. However, there is still a significant change of the aromatic signals from the azidobenzene group multiplet (in the starting material) to well-resolved doublets and triplets of the annulated benzene ring (in the product) that are characteristic for the cyclization of all Ugi products (Figure 5).

This NMR pattern change can be used as the only remarkable indicator of successful click reaction on non-terminal alkynes.

Increasing the temperature allowed higher yields of the product, exactly as it did when terminal alkynes were used as reactants (Table 1, entries 9 and 10). The investigation on the optimization of the reaction parameters to find safer and ecologically benign conditions included temperature, solvent, and reaction time (Table 1, entries 11–24) as variables. Reactions in such solvents as ethanol and glycerol resulted in almost no conversion. The treatment in water led to a mixture of the product with the starting material where low conversion can be explained by solubility issues. To overcome this, the reaction was carried out in an acetonitrile/water (4:1) mixture similar to the procedure reported by Y. Xia et al. [23] that allowed the isolation of the target compound in 50% yield. The temperature was proved to play a key role in the cyclization, however, above 150 °C conversion decreased due to significant thermal decomposition of the substrate and/or the product (Table 1, entries 15–17). The varying acetonitrile/water ratio demonstrated no impact on the product yield (Table 1, entries 18 and 19). The reaction was found to proceed well in pure acetonitrile as well (Table 1, entries 20–22) and even led to a better yield at 140 °C than in the acetonitrile/water mixture. Finally, we tested NMP as a green solvent that is also one of the safest choices for performing high-temperature MW-assisted syntheses (high boiling point, nonvolatile). Even though it brings difficulties towards product isolation resulting in material loss, the reaction time can be reduced to 30 minutes at 140 °C, and the target compound can be obtained in 77% yield (Table 1, entry 24). Further decreasing of reaction holding time to 15 minutes is not possible due to observing only partial conversion of starting material by TLC.

**Table 1:** Optimizing the reaction conditions for IAAC on internal alkynes.

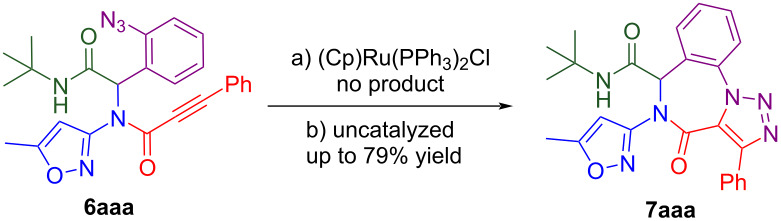				

**Part A.** Ru-catalyzed conditions screening^a^.				

Entry	Solvent	Temperature, °C	Reaction time, h	Yield, %

1	DCM	rt	48	unidentified mixture
2	DCM	−15	96	NR
3	DCM	4	72	NR
4	DCM	40	48	unidentified mixture
5	PhH	rt	48	unidentified mixture
6	THF	55	8	traces^b^
7	PhH	78	6	traces^b^

**Part B.** Uncatalyzed condition screening.				

8	PhH	reflux	12	35
9	PhH	120/MW	2	68
10	PhH	140/MW	1	77
11	EtOH	reflux	12	traces
12	EtOH	100/MW	1	traces
13	glycerol	120/MW	2	NR
14	H_2_O	120/MW	2	**6aaa** + **7aaa** mixture
15	MeCN/H_2_O (4:1)	120/MW	2	50
16	MeCN/H_2_O (4:1)	140/MW	1	60
17	MeCN/H_2_O (4:1)	150/MW	1	51
18	MeCN/H_2_O (2:1)	140/MW	1	60
19	MeCN/H_2_O (1:1)	140/MW	1	64
20	MeCN	120/MW	2	39
21	MeCN	140/MW	1	76
22	MeCN	150/MW	1	51
23	NMP	140/MW	1	79
24	NMP	140/MW	0.5	77

^a^Reaction conditions: 0.22 mmol scale, 10 mol % catalyst loading, inert atmosphere; ^b^new spot observed on TLC; NR = no reaction; MW = microwave irradiation; US = ultrasonic irradiation.

Then our optimal conditions were tested on other Ugi substrates which contain a fragment of the internal alkyne with controlling reaction time by TLC (Scheme 5). In general, 0.5–1 hour reaction time and 140 °C were enough to achieve full substrate transformation to the product (by TLC), but for Ugi products **6aba, 6aca** and **6bba**, based on aromatic amines, a higher temperature was required. In contrast to IAAC on terminal alkynes, the yields for the cyclization of internal alkynes were significantly lower due to the abovementioned difficulties with isolation and thermal degradation.

## Conclusion

In conclusion, we have advanced the creation of a convenient tandem approach to potentially biologically active 1,2,3-triazolobenzodiazepinones. The developed synthetic procedure includes a four-component Ugi reaction followed by microwave-assisted intramolecular azide–alkyne cycloaddition and allows to build a complex scaffold in just two steps. Unusually, the azide–alkyne cycloaddition for both terminal and non-terminal alkynes was effective under catalyst-free conditions while normally for non-terminal alkynes a ruthenium-based catalysis is required. Using the developed method, a library of 22 target compounds was obtained. Currently, we are working on testing some library representatives for their biological activity.

## Supporting Information

File 1 General synthetic procedures, characteristics of compounds **6** and **7**, X-ray experimental data and copies of ^1^H and ^13^C NMR spectra.
