# Supplementary material for: Effective microwave-assisted approach to 1,2,3-triazolobenzodiazepinones via tandem Ugi reaction/catalyst-free intramolecular azide–alkyne cycloaddition
Source: Beilstein J Org Chem. 2021 Mar 8;17:678–87. doi: 10.3762/bjoc.17.57 (PMC7961865; doi:10.3762/bjoc.17.57)

BEILSTEIN JOURNAL OF ORGANIC CHEMISTRY

Supporting Information

for

Effective microwave-assisted approach to 1,2,3-triazolobenzodiazepinones via tandem Ugi reaction/catalyst-free intramolecular azide–alkyne cycloaddition

Maryna O. Mazur, Oleksii S. Zhelavskyi, Eugene M. Zviagin, Svitlana V. Shishkina, Vladimir I. Musatov, Maksim A. Kolosov, Elena H. Shvets, Anna Yu. Andryushchenko and Valentyn A. Chebanov

Beilstein J. Org. Chem. **2021,** *17,* 678–687. doi:10.3762/bjoc.17.57

General synthetic procedures, characteristics of compounds 6 and 7, X-ray experimental data and copies of ^{1}H and ^{13}C NMR spectra

License and Terms: This is a supporting information file under the terms of the Creative Commons Attribution License (https://creativecommons.org/licenses/by/4.0). Please note that the reuse, redistribution and reproduction in particular requires that the author(s) and source are credited and that individual graphics may be subject to special legal provisions.
The license is subject to the *Beilstein Journal of Organic Chemistry* terms and conditions: (https://www.beilstein-journals.org/bjoc/terms)

Table of contents

1. General synthetic aspects and procedures S2
2. Characteristics of compounds **6** and **7** S3
3. X-ray experimental data S15
4. ^{1}H and ^{13}C NMR spectra S16

1. General synthetic aspects and procedures

^{1}H and ^{13}C NMR spectra were acquired on a Varian Mercury VX-400 instrument at 400 and 100 MHz, respectively, in DMSO-d_6. Experiments under microwave irradiation were carried out by using an Anton Paar Monowave 300 reactor (monomode cavity, 2.45 GHz).

Synthesis of 2-azidobenzaldehyde 2. 2-nitrobenzaldehyde (**1**, 33.1 mmol, 5 g) and sodium azide (99.3 mmol, 6.5 g) are placed in the round bottom flask equipped with a condenser and 40 ml of dry DMF are added. The reaction mixture is stirred on the oil bath (55 °C) for 7–10 days. After that the reaction mixture is poured on ice and extracted by hexane (4 × 20 ml). The organic layers were combined, washed with brine, dried over Na_2SO_4 and the solvent was removed. The obtained yellow oil was purified by column chromatography. The product was stored at 4 °C in a freezer. Yield 3.37 g (69%), yellow crystals, mp 33-35°C. ^{1}H NMR (400 MHz, DMSO-d_6) δ (ppm): 10.16 (s, 1H), 7.78 – 7.61 (m, 2H), 7.41 (d, *J* = 8.1 Hz, 1H), 7.26 (t, *J* = 7.5 Hz, 1H).

General procedure for the syntheses of compounds 6 in MeOH (Ugi reaction). Amine **5** (2 mmol) and 2-azidobenzaldehyde (**2**, 2 mmol, 294 mg) were placed in a capped 10 ml vial and 2 ml of MeOH are added. The precipitate of intermediate imine can form. The reaction mixture was stirred at rt for 2 hours, then carboxylic acid **3** (2 mmol) was added and the reaction was stirred for an additional 20 minutes. After that isocyanide **4** (2 mmol) was added dropwise, the vial was hermetically closed with a cap and stirred overnight. The formed precipitate of Ugi product **6** was filtered out, washed with a minimum quantity of MeOH to remove red color (if necessary), and air-dried.

General procedure for the syntheses of compounds 6ada and 6aea in DCM (Ugi reaction). Amine **5** (2 mmol), 2-azidobenzaldehyde (**2**, 2 mmol, 294 mg), carboxylic acid **3** (2 mmol) and isocyanide **4** (2 mmol) were placed in the round bottom flask and 16 ml of DCM were added. The reaction mixture was stirred on the oil bath (40 °C) for 2–3 days. After that the solvent was removed from the reaction mixture using a rotary evaporator. To the crude product the minimum amount of MeOH is added. After stirring for 15–20 minutes the precipitate of Ugi product **6** is filtered out and air-dried.

General procedure for the syntheses of compounds 7.
Method A. The Ugi product **6** (100 mg) was placed in a G10 microwave vial. 3 ml of NMP was added. The vial was inserted in the microwave reactor and irradiated while stirring. The reaction progress was monitored by TLC (EtOAc/hexane = 1:2). After the reaction was completed and the reaction mixture was poured on ice a precipitate was formed. The precipitate was removed by filtration and dried in the air.

Method B. The Ugi product **6** (100 mg) was placed in a G10 microwave vial. 3 ml of toluene was added. The vial was inserted in the microwave reactor and irradiated while stirring. The reaction progress was monitored by TLC (EtOAc/hexane = 1:2). After the reaction was completed the reaction mixture was cooled. Compounds **7aab**, **7abb, 7aeb** were isolated by removing the toluene in vacuo. All compounds were dried in the air.

2. Characteristics of compounds 6 and 7

6aaa

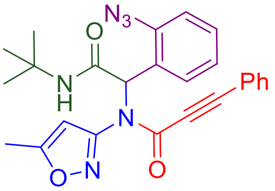

***N*-(1-(2-Azidophenyl)-2-(*tert*-butylamino)-2-oxoethyl)-*N*-(5-methylisoxazol-3-yl)-3-phenylpropiolamide (6aaa).**
White solid.
Yield 57%, mp. 165-167 °C;
Elemental analysis calcd for $C_{25}H_{24}N_6O_3$: C 65.78, H 5.30, N 18.41, found: C 65.81, H 5.67, N 18.31.
MS (EI, 70 eV): *m*/*z* = 457.20 $[M]^+$.
^{1}H NMR (400 MHz, DMSO-d_6) δ 8.03 (s, 1H, NH), 7.03-7.60 (m, 9H, ArH), 6.40 (s, 1H, CH), 6.19 (s, 1H, CH), 2.29 (s, 3H, CH_3), 1.26 (s, 9H, *t*-Bu).
^{13}C NMR (100 MHz, DMSO-d_6) δ 171.07, 167.46, 160.18, 152.91, 139.64, 132.63, 131.31, 130.63, 130.34, 129.40, 126.10, 125.05, 119.48, 119.21, 103.58, 90.60, 82.12, 58.50, 51.13, 28.72, 12.49.

6aba

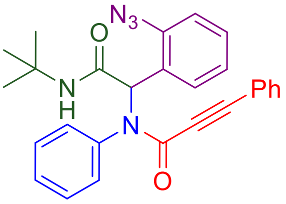

***N*-(1-(2-Azidophenyl)-2-(*tert*-butylamino)-2-oxoethyl)-*N*,3-diphenylpropiolamide (6aba).**
Rotamer ratio: 90:10.
White solid.
Yield 67%, mp. 184-186 °C;
Elemental analysis calcd for $C_{27}H_{25}N_5O_2$: C 71.82, H 5.58, N 15.51, found: C 72.25, H 5.86, N 15.34.
MS (EI, 70 eV): *m*/*z* = 452.20 $[M]^+$.
^{1}H NMR (400 MHz, DMSO-d_6) δ 8.11 (s, 0.1H, NH), 7.90 (s, 0.9H, NH), 6.85 – 7.81 (m, 14H, ArH), 6.42 (s, 0.1H, CH), 6.19 (s, 0.9H, CH), 1.27 (s, 8.1H, *t*-Bu), 1.19 (s, 0.9H, *t*-Bu).
^{13}C NMR (100 MHz, DMSO-d_6) δ 168.41, 153.64, 139.39, 139.29, 132.31, 130.86, 130.83, 130.25, 129.25, 128.44, 126.58, 124.97, 119.86, 118.90, 90.60, 83.31, 59.42, 50.97, 28.81.

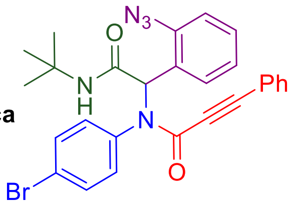

6aca

***N*-(1-(2-Azidophenyl)-2-(*tert*-butylamino)-2-oxoethyl)-*N*-(4-bromophenyl)-3-phenylpropiolamide (6aca).**

Rotamer ratio: 80:20.

White solid.

Yield 56%, mp. 178-180 °C;

Elemental analysis calcd for $C_{27}H_{24}BrN_5O_2$: C 61.14, H 4.56, N, 13.20, found: C 61.33, H 4.36, N 13.06.

MS (EI, 70 eV): *m*/*z* = 429.95 $[C_{19}H_{19}BrN_5O_2]^+$.

^{1}H NMR (400 MHz, DMSO-d_6) δ 8.19 (s, 0.2H, NH), 7.98 (s, 0.8H, NH), 6.87 – 7.83 (m, 13H, ArH), 6.42 (s, 0.2H, CH), 6.19 (s, 0.8H, CH), 1.27 (s, 7.4H, *t*-Bu), 1.19 (s, 1.6H, *t*-Bu).

^{13}C NMR (100 MHz, DMSO-d_6) δ 168.35, 153.39, 139.30, 138.77, 133.02, 132.26, 131.49, 131.06, 130.71, 130.49, 129.34, 126.17, 125.10, 121.61, 119.64, 119.01, 91.05, 83.04, 59.33, 51.04, 28.77.

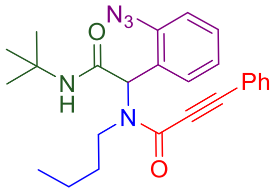

6ada

***N*-(1-(2-Azidophenyl)-2-(*tert*-butylamino)-2-oxoethyl)-*N*-butyl-3-phenylpropiolamide (6ada).**

Rotamer ratio: 50:50.

White solid.

Yield 40%, mp. 156-158 °C;

Elemental analysis calcd for $C_{25}H_{29}N_5O_2$: C 69.58, H 6.77, N 16.23, found: C 70.03, H 7.14, N 15.97.

MS (EI, 70 eV): *m*/*z* = 432.15 $[M]^+$.

^{1}H NMR (400 MHz, DMSO-d_6) δ 8.05 (s, 0.5H, NH), 7.86 (s, 0.5H, NH), 7.2 – 7.72 (m, 9H, ArH), 6.17 (s, 0.5H, CH), 6.04 (s, 0.5H, CH), 3.37 – 3.54 (m, 1H, *n*-Bu NCH_2), 2.98 – 3.08 (m, 0.5H, *n*-Bu NCH_2), 1.33 – 1.43 (m, 0.5H, *n*-Bu NCH_2), 1.22 and 1.25 (both singlets, 9H, *t*-Bu), 0.52 – 1.13 (m, 7H, *n*-Bu CH_2 + *n*-Bu CH_3).

^{13}C NMR (100 MHz, DMSO-d_6) δ 168.60, 168.43, 154.80, 154.25, 139.79, 139.74, 132.98, 132.47, 130.96, 130.94, 130.68, 130.53, 130.26, 130.11, 129.47, 129.21, 127.58, 127.51, 125.66, 125.56, 120.31, 120.12, 119.37, 89.69, 88.95, 82.77, 82.50, 60.87, 56.50, 50.94, 50.92, 47.04, 44.46, 32.06, 30.01, 28.75, 28.66, 28.16, 19.93, 19.84, 13.79.

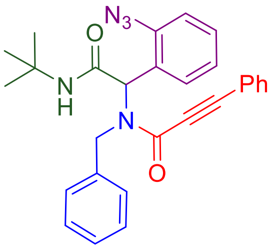

6aea

***N*-(1-(2-Azidophenyl)-2-(*tert*-butylamino)-2-oxoethyl)-*N*-benzyl-3-phenylpropiolamide (6aea).**
Rotamer ratio: 55:45.
Pale yellow solid.
Yield 55%, mp. 169-171 °C;
Elemental analysis calcd for $C_{28}H_{27}N_5O_2$: C 72.24, H 5.85, N 15.04, found: C 72.08, H 6.19, N 14.90.
MS (EI, 70 eV): *m*/*z* = 466.15 $[M]^+$.
^{1}H NMR (400 MHz, DMSO-*d*$_6$) δ 8.10 (s, 0.55H, NH), 7.90 (s, 0.45H, NH), 6.67 – 7.77 (m, 14H, ArH), 6.19 (s, 0.55H, CH), 6.06 (s, 0.45H, CH), 5.18 (d, *J* = 17.1 Hz, 0.5H, benzyl CH_2), 5.07 (d, *J* = 15.6 Hz, 0.5H, benzyl CH_2), 4.46 (d, *J* = 17.1 Hz, 0.5H, benzyl CH_2), 3.98 (d, *J* = 15.7 Hz, 0.5H, benzyl CH_2), 1.21 and 1.23 (both singlets, 9H, *t*-Bu).
^{13}C NMR (100 MHz, DMSO-*d*$_6$) δ 168.82, 168.58, 155.77, 155.12, 139.80, 139.61, 138.16, 137.84, 133.18, 132.52, 131.21, 131.00, 130.63, 130.42, 130.24, 130.07, 129.35, 129.26, 127.84, 127.68, 127.23, 126.70, 126.62, 126.47, 125.40, 125.24, 120.11, 119.85, 118.68, 118.57, 90.86, 89.63, 82.88, 82.15, 60.54, 56.39, 50.94, 47.79, 28.73, 28.60.

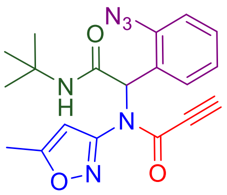

6aab

***N*-(1-(2-Azidophenyl)-2-(*tert*-butylamino)-2-oxoethyl)-*N*-(5-methylisoxazol-3-yl)propiolamide (6aab).**
White solid.
Yield 44%, mp. 164-166 °C;
Elemental analysis calcd for $C_{19}H_{20}N_6O_3$: C 59.99, H 5.30, N 22.09, found: C 60.05, H 5.56, N 21.88.
MS (EI, 70 eV): *m*/*z* = 381.05 $[M]^+$.
^{1}H NMR (400 MHz, DMSO-*d*$_6$) δ 7.99 (s, 1H, NH), 7.00 – 7.39 (m, 4H, ArH), 6.33 (s, 1H, CH), 6.12 (s, 1H, CH), 4.40 (s, 1H, alkyne CH), 2.26 (s, 3H, CH_3), 1.26 (s, 9H, *t*-Bu).
^{13}C NMR (100 MHz, DMSO-*d*$_6$) δ 171.26, 167.30, 159.84, 152.12, 139.60, 130.73, 130.24, 125.72, 125.04, 119.25, 103.54, 83.48, 76.01, 58.30, 51.12, 28.65, 12.52.

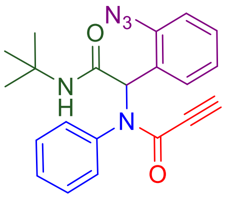

6abb

***N*-(1-(2-Azidophenyl)-2-(*tert*-butylamino)-2-oxoethyl)-*N*-phenylpropiolamide (6abb).**
Rotamer ratio: 90:10.
White solid.
Yield 53%, mp. 174-176 °C;
Elemental analysis calcd for $C_{21}H_{21}N_5O_2$: C 67.18, H 5.64, N 18.65, found: C 67.35, H 6.07, N 18.46.
MS (EI, 70 eV): *m*/*z* = 376.05 $[M]^+$.
^{1}H NMR (400 MHz, DMSO-d_6) δ 7.96 (s, 0.1H, NH), 7.87 (s, 0.9H, NH), 6.86 – 7.42 (m, 9H, ArH), 6.27 (s, 0.1H, CH), 6.10 (s, 0.9H, CH), 4.74 (s, 0.1H, triazole CH), 4.14 (s, 0.9H, triazole CH), 1.28 and 1.25 (both singlets, 9H, *t*-Bu).
^{13}C NMR (100 MHz, DMSO-d_6) δ 167.90, 152.54, 138.89, 138.58, 130.43, 130.29, 129.89, 128.17, 128.12, 125.99, 124.54, 118.47, 83.01, 76.81, 59.02, 50.58, 28.46.

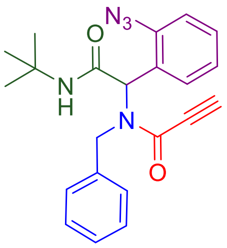

6aeb

***N*-(1-(2-Azidophenyl)-2-(*tert*-butylamino)-2-oxoethyl)-*N*-benzylpropiolamide (6aeb).**
Rotamer ratio: 55:45.
Pale orange solid.
Yield 67%, mp. 178-180 °C;
Elemental analysis calcd for $C_{22}H_{23}N_5O_2$: C 67.85, H 5.95, N 17.98, found: C 68.05, H 6.23, N 17.65.
MS (EI, 70 eV): *m*/*z* = 390.10 $[M]^+$.
^{1}H NMR (400 MHz, DMSO-d_6) δ 7.98 (s, 0.45H, NH), 7.85 (s, 0.55H, NH), 6.63 – 7.31 (m, 9H, ArH), 6.07 (s, 0.45H, CH), 5.96 (s, 0.55H, CH), 5.10 (d, *J* = 16.9 Hz, 0.55H, benzyl CH_2), 4.98 (d, *J* = 15.7 Hz, 0.45H, benzyl CH_2), 4.67 (s, 0.45H, triazole CH), 4.51 (s, 0.55H, triazole CH), 4.38 (d, *J* = 17.0 Hz, 0.45H, benzyl CH_2), 3.97 (d, *J* = 15.7 Hz, 0.55H, benzyl CH_2), 1.21 and 1.25 (both singlets, 9H, *t*-Bu).
^{13}C NMR (100 MHz, DMSO-d_6) δ 168.43, 168.40, 155.05, 154.45, 139.72, 139.58, 137.94, 137.34, 130.57, 130.42, 130.20, 130.02, 127.74, 127.69, 127.12, 126.74, 126.66, 126.56, 126.46, 125.34, 125.17, 118.77, 118.44, 83.70, 82.48, 76.99, 76.23, 60.50, 56.23, 51.08, 50.92, 47.60, 28.71.

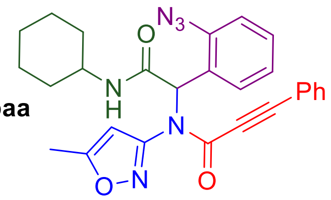

6baa

***N*-(1-(2-Azidophenyl)-2-(cyclohexylamino)-2-oxoethyl)-*N*-(5-methylisoxazol-3-yl)-3-phenylpropiolamide (6baa).**
White solid.
Yield 25%, mp. 149-151 °C;
Elemental analysis calcd for $C_{27}H_{26}N_6O_3$: C 67.21, H 5.4, N 17.42, found: C 67.06, H 5.92, N 17.09.
MS (EI, 70 eV): *m*/*z* = 483.15 $[M]^+$.
^{1}H NMR (400 MHz, DMSO-d_6) δ 7.00 – 8.28 (m, 10H, ArH + NH), 6.40 (s, 1H, CH), 6.22 (s, 1H, CH), 3.57 – 3.69 (m, 1H, cyclohexyl NCH), 2.30 (s, 3H, CH_3), 0.99 – 1.80 (m, 10H, cyclohexyl CH_2).
^{13}C NMR (100 MHz, DMSO-d_6) δ 171.33, 167.06, 160.14, 152.84, 139.56, 132.64, 131.43, 130.79, 130.44, 125.60, 125.12, 119.32, 119.27, 103.65, 90.58, 81.97, 57.97, 53.90, 48.54, 32.52, 25.54, 24.77, 12.56.

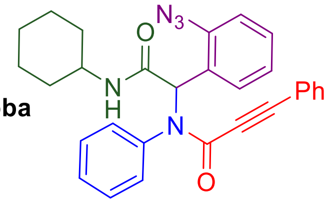

6bba

***N*-(1-(2-Azidophenyl)-2-(cyclohexylamino)-2-oxoethyl)-*N*,3-diphenylpropiolamide (6bba).**
Rotamer ratio: 85:15.
White solid.
Yield 75%, mp. 160-162 °C;
Elemental analysis calcd for $C_{29}H_{27}N_5O_2$: C 72.94, H 5.70, N 14.66, found: C 72.45, H 6.01, N 14.49.
MS (EI, 70 eV): *m*/*z* = 478.20 $[M]^+$.
^{1}H NMR (400 MHz, DMSO-d_6) δ 8.12, 6.81 – 8.17 (m, 15H, ArH + NH), 6.46 (s, 0.15H, CH), 6.23 (s, 0.85H, CH), 3.55 – 3.72 (m, 1H, cyclohexyl NCH), 0.95 – 1.83 (m, 10H, cyclohexyl CH_2).
^{13}C NMR (100 MHz, DMSO-d_6) δ 168.03, 153.70, 139.32, 139.28, 132.33, 131.03, 130.88, 130.35, 129.24, 128.48, 126.27, 124.97, 119.82, 118.88, 90.67, 83.25, 58.99, 48.54, 32.54, 25.59, 24.99, 24.85.

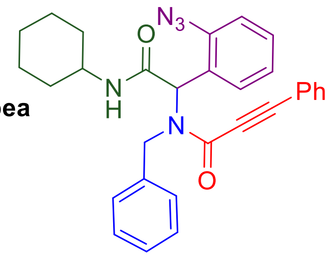

6bea

N-(1-(2-Azidophenyl)-2-(cyclohexylamino)-2-oxoethyl)-N-benzyl-3-phenylpropiolamide (6bea).

Rotamer ratio: 50:50.

Pale yellow solid.

Yield 51%, mp. 163-165 °C;

Elemental analysis calcd for $C_{30}H_{29}N_5O_2$: C 73.30, H 5.95, N 14.25, found: C 73.52, H 6.40, N 13.96.

MS (EI, 70 eV): *m/z* = 492.15 $[M]^+$.

^{1}H NMR (400 MHz, DMSO-d_6) δ 6.68 – 8.33 (m, 15H, ArH + NH), 6.24 (s, 0.5H, CH), 6.08 (s, 0.5H, CH), 5.19 (d, *J* = 17.0 Hz, 0.5H, benzyl CH_2), 5.07 (d, *J* = 15.6 Hz, 0.5H, benzyl CH_2), 4.47 (d, *J* = 17.1 Hz, 0.5H, benzyl CH_2), 4.04 (d, *J* = 15.7 Hz, 0.5H, benzyl CH_2), 3.52 – 3.69 (m, 1H, cyclohexyl NCH), 0.96 – 1.8 (m, 10H, cyclohexyl CH_2).

^{13}C NMR (100 MHz, DMSO-d_6) δ 168.25, 168.10, 155.61, 155.17, 139.73, 139.59, 138.10, 137.78, 133.09, 132.53, 131.20, 131.03, 130.69, 130.53, 130.40, 130.14, 129.35, 129.28, 127.87, 127.71, 127.25, 126.75, 126.61, 126.51, 126.48, 126.39, 125.42, 125.27, 90.92, 89.73, 82.82, 82.04, 60.44, 56.20, 50.97, 48.34, 48.22, 47.66, 32.53, 32.50, 32.43, 32.37, 25.56, 25.52, 24.93, 24.81, 24.75, 24.71.

7aaa

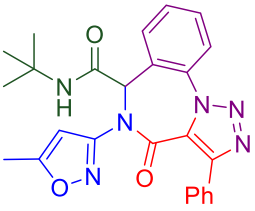

N-(tert-Butyl)-5-(5-methylisoxazol-3-yl)-4-oxo-3-phenyl-5,6-dihydro-4H-benzo[f][1,2,3]triazolo[1,5-a][1,4]diazepine-6-carboxamide (7aaa).

White solid.

Yield 79%, mp. 247-249 °C;

Elemental analysis calcd for $C_{25}H_{24}N_6O_3$: C 65.78, H 5.30, N 18.41, found: C 65.55, H 5.73, N 18.11.

MS (EI, 70 eV): *m/z* = 457.15 $[M]^+$.

^{1}H NMR (400 MHz, DMSO-d_6) δ 7.43 – 8.06 (m, 6H, ArH), 7.11 (s, 1H, NH), 6.79 (s, 1H, CH), 6.46 (s, 1H, CH), 2.41 (s, 3H, CH_3), 0.91 (s, 9H, *t*-Bu).

^{13}C NMR (100 MHz, DMSO-d_6) δ 171.61, 164.99, 160.23, 157.72, 150.04, 134.48, 132.59, 131.33, 130.30, 129.93, 129.85, 129.46, 128.82, 128.58, 128.43, 123.54, 98.54, 62.30, 51.80, 28.12, 12.61.

7aba

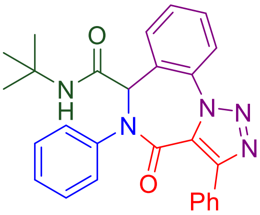

N-(tert-Butyl)-4-oxo-3,5-diphenyl-5,6-dihydro-4H-benzo[f][1,2,3]triazolo[1,5-a][1,4]diazepine-6-carboxamide (7aba).

Gray solid.

Yield 66%, mp. >250°C;

Elemental analysis calcd for $C_{27}H_{25}N_5O_2$: C 71.82, H 5.58, N 15.51, found: C 70.60, H 5.64, N 14.78.

MS (EI, 70 eV): *m/z* = 423.05 $[C_{27}H_{25}N_3O_2]^+$.

^{1}H NMR (400 MHz, DMSO-d_6) δ 7.26 – 8.15 (m, 14H, ArH), 6.35 (s, 1H, NH), 5.82 (s, 1H, CH), 0.92 (s, 9H, *t*-Bu).

^{13}C NMR (100 MHz, DMSO-d_6) δ 166.12, 158.64, 149.36, 142.89, 134.24, 132.00, 131.25, 131.03, 130.48, 130.27, 129.67, 129.28, 129.15, 128.73, 128.63, 127.90, 127.07, 123.70, 67.33, 51.75, 28.20.

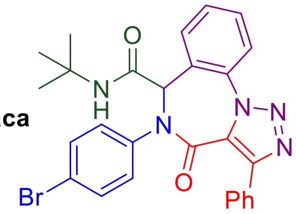

5-(4-Bromophenyl)-*N*-(*tert*-butyl)-4-oxo-3-phenyl-5,6-dihydro-4*H*-benzo[*f*][1,2,3]triazolo[1,5-*a*][1,4]diazepine-6-carboxamide (7aca).
Grey solid.
Yield 50%, mp. >250 °C;
Elemental analysis calcd for $C_{27}H_{24}BrN_5O_2$: C 61.14, H 4.56, N 13.20, found: C 60.86, H 4.68, N 13.56.
MS (EI, 70 eV): *m*/*z* = 502.95 $[C_{27}H_{24}BrN_3O_2]^+$.
^{1}H NMR (400 MHz, DMSO-d_6) δ 7.24 – 8.12 (m, 13H, ArH), 6.41 (s, 1H, NH), 5.86 (s, 1H, CH), 0.92 (s, 9H, *t*-Bu).
^{13}C NMR (100 MHz, DMSO-d_6) δ 166.07, 158.63, 149.50, 141.95, 134.25, 132.55, 132.02, 131.30, 130.77, 130.49, 130.17, 129.39, 129.20, 129.01, 128.74, 128.63, 123.71, 120.62, 67.02, 51.82, 28.20.

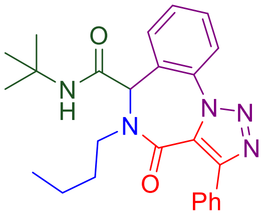

***N*-(*tert*-Butyl)-5-butyl-4-oxo-3-phenyl-5,6-dihydro-4*H*-benzo[*f*][1,2,3]triazolo[1,5-*a*][1,4]diazepine-6-carboxamide (7ada).**
White solid.
Yield 86%, mp. 203-205 °C;
Elemental analysis calcd for $C_{25}H_{29}N_5O_2$: C 69.58, H 6.77, N 16.23, found: C 67.92, H 6.95, N 16.49.
MS (EI, 70 eV): *m*/*z* = 432.10 $[M]^+$.
^{1}H NMR (400 MHz, DMSO-d_6) 7.38 – 8.05 (m, 9H, ArH), 6.84 (s, 1H, NH), 5.64 (s, 1H, CH), 3.95 – 4.07 (m, 1H, *n*-Bu NCH_2), 3.39 – 3.50 (m, 1H, *n*-Bu NCH_2), 0.69 – 1.69 (m, 16H, *t*-Bu + *n*-Bu CH_3 + *n*-Bu CH_2).
^{13}C NMR (100 MHz, DMSO-d_6) δ 166.37, 158.53, 148.29, 134.39, 132.80, 131.51, 130.55, 130.21, 129.99, 129.09, 129.04, 128.67, 128.41, 123.27, 63.72, 51.51, 49.04, 30.00, 28.16, 19.61, 13.94.

7aea

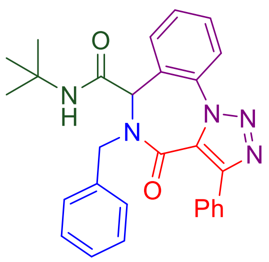

5-Benzyl-*N*-(*tert*-butyl)-4-oxo-3-phenyl-5,6-dihydro-4*H*-benzo[*f*][1,2,3]triazolo[1,5-*a*][1,4]diazepine-6-carboxamide (7aea).
White solid.
Yield 82%, mp. 191-193 °C;
Elemental analysis calcd for $C_{28}H_{27}N_5O_2$: C 72.24, H 5.85, N 15.04, found: C 72.35, H 6.33, N 15.08.
MS (EI, 70 eV): *m*/*z* = 437.05 [$C_{28}H_{27}N_3O_2$]$^+$.
^{1}H NMR (400 MHz, DMSO-d_6) δ 7.2 – 7.99 (m, 14H, ArH), 6.65 (s, 1H, NH), 5.60 (s, 1H, CH), 5.17 (d, *J* = 14.6 Hz, 1H, CH_2), 4.66 (d, *J* = 14.7 Hz, 1H, CH_2), 0.84 (s, 9H, *t*-Bu).
^{13}C NMR (100 MHz, DMSO-d_6) δ 166.11, 158.87, 148.42, 137.28, 134.21, 132.13, 131.46, 130.36, 130.02, 129.62, 129.28, 128.92, 128.90, 128.79, 128.58, 128.37, 128.13, 123.16, 64.22, 52.61, 51.41, 28.02.

7aab

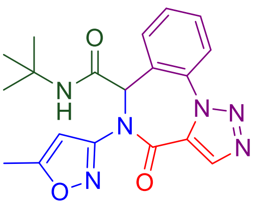

***N*-(*tert*-Butyl)-5-(5-methylisoxazol-3-yl)-4-oxo-5,6-dihydro-4*H*-benzo[*f*][1,2,3]triazolo[1,5-*a*][1,4]diazepine-6-carboxamide (7aab).**
White solid.
Yield >99%, mp. 170-172 °C;
Elemental analysis calcd for $C_{19}H_{20}N_6O_3$: C 59.99, H 5.30, N 22.09, found: C 59.68, H 5.07, N 21.68.
MS (EI, 70 eV): *m*/*z* = 381.10 [M]$^+$.
^{1}H NMR (400 MHz, DMSO-d_6) δ 8.48 (s, 1H, triazole CH), 7.58 – 7.98 (m, 4H, ArH) 6.92 (s, 1H, NH), 6.76 (s, 1H, CH), 6.41 (s, 1H, CH), 2.43 (s, 3H, CH_3), 0.95 (s, 9H, *t*-Bu).
^{13}C NMR (100 MHz, DMSO-d_6) δ 171.51, 165.00, 160.67, 157.32, 134.61, 133.84, 132.84, 131.34, 130.26, 128.90, 123.41, 98.67, 62.70, 55.33, 51.53, 28.18, 12.66.

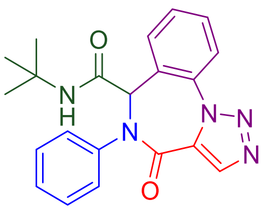

7abb

***N*-(*tert*-Butyl)-4-oxo-5-phenyl-5,6-dihydro-4*H*-benzo[*f*][1,2,3]triazolo[1,5-*a*][1,4]diazepine-6-carboxamide (7abb).**
White solid.
Yield >99%, mp. 110-112 °C;
Elemental analysis calcd for $C_{21}H_{21}N_5O_2$: C 67.18, H 5.64, N 18.65, found: C 65.99, H 5.92, N 18.43.
MS (EI, 70 eV): *m*/*z* = 376.10 $[M]^+$.
^{1}H NMR (400 MHz, DMSO-d_6) δ 8.36 (s, 1H, triazole CH), 7.23 – 8.04 (m, 9H, ArH), 6.52 (s, 1H, NH), 5.69 (s, 1H, CH), 0.97 (s, 9H, *t*-Bu).
^{13}C NMR (100 MHz, DMSO-d_6) δ 166.19, 158.22, 143.46, 138.50, 134.42, 134.35, 132.15, 131.24, 130.45, 129.82, 129.76, 128.05, 127.09, 123.51, 67.60, 51.51, 28.20.

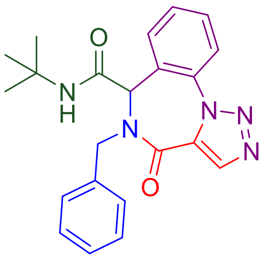

7aeb

5-Benzyl-*N*-(*tert*-butyl)-4-oxo-5,6-dihydro-4*H*-benzo[*f*][1,2,3]triazolo[1,5-*a*][1,4]diazepine-6-carboxamide (7aeb).
Light brown solid.
Yield >99%, mp. 137-139 °C;
Elemental analysis calcd for $C_{22}H_{23}N_5O_2$: C 67.85, H 5.95, N 17.98, found: C 67.50, H 6.14, N 18.03.
MS (EI, 70 eV): *m*/*z* = 390.10 $[M]^+$.
^{1}H NMR (400 MHz, DMSO-d_6) δ 8.35 (s, 1H, triazole CH), 7.21 – 8.01 (m, 9H, ArH), 6.41 (s, 1H, NH), 5.47 (s, 1H, CH), 4.96 (d, *J* = 14.7 Hz, 1H, CH_2), 4.75 (d, *J* = 14.7 Hz, 1H, CH_2), 0.86 (s, 9H, *t*-Bu).
^{13}C NMR (100 MHz, DMSO-d_6) δ 165.96, 158.35, 138.10, 137.06, 134.49, 134.03, 131.86, 130.62, 130.47, 129.81, 128.95, 128.93, 128.12, 123.12, 64.42, 52.51, 51.16, 28.12.

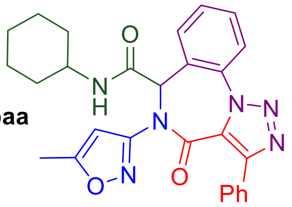

N-Cyclohexyl-5-(5-methylisoxazol-3-yl)-4-oxo-3-phenyl-5,6-dihydro-4H-benzo[f][1,2,3]triazolo[1,5-a][1,4]diazepine-6-carboxamide (7baa).
Gray solid.
Yield 58%, mp. 229-231 °C;
Elemental analysis calcd for $C_{27}H_{26}N_6O_3$: C 67.21, H 5.43, N 17.42, found: C 67.03, H 5.64, N 17.29.
MS (EI, 70 eV): *m/z* = 483.20 $[M]^+$.
^{1}H NMR (400 MHz, DMSO-d_6) 7.39 – 8.06 (m, 10H, ArH + NH), 6.78 (s, 1H, CH), 6.49 (s, 1H, CH), 3.18 – 3.31 (m, 1H, cyclohexyl CH), 2.41 (s, 3H, CH_3), 0.76 – 1.5 (m, 10H, cyclohexyl CH_2).
^{13}C NMR (100 MHz, DMSO-d_6) δ 171.60, 164.71, 160.22, 157.67, 150.03, 134.60, 132.58, 131.38, 130.37, 129.85, 129.61, 129.44, 128.78, 128.27, 123.61, 98.57, 62.09, 49.32, 32.03, 31.69, 25.39, 24.99, 12.63.

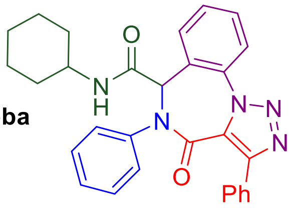

N-Cyclohexyl-4-oxo-3,5-diphenyl-5,6-dihydro-4H-benzo[f][1,2,3]triazolo[1,5-a][1,4]diazepine-6-carboxamide (7bba).
Grey solid.
Yield 46%, mp. 229-231 °C;
Elemental analysis calcd for $C_{29}H_{27}N_5O_2$: C 72.94, H 5.70, N 14.66, found: C 72.77, H 5.99, N 14.20.
MS (EI, 70 eV): *m/z* = 478.20 $[M]^+$.
^{1}H NMR (400 MHz, DMSO-d_6) δ 7.12 – 8.15 (m, 15H, ArH + NH), 5.86 (s, 1H, CH), 0.74 – 1.57 (m, 10H, cyclohexyl CH_2), cycloxehyl NCH under water.
^{13}C NMR (100 MHz, DMSO-d_6) δ 165.90, 158.64, 149.23, 142.91, 134.37, 132.03, 131.15, 130.87, 130.43, 130.30, 129.60, 129.16, 129.12, 128.71, 128.68, 127.85, 127.12, 66.99, 49.28, 31.98, 31.68, 25.38, 25.03.

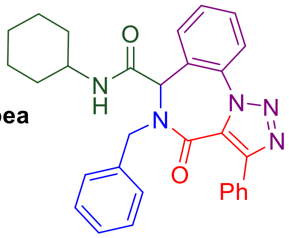

7bea

5-Benzyl-*N*-cyclohexyl-4-oxo-3-phenyl-5,6-dihydro-4*H*-benzo[*f*][1,2,3]triazolo[1,5-*a*][1,4]diazepine-6-carboxamide (7bea).

This compound has been already described in the literature [1].

White solid.

Yield 68%, mp. 195-197 °C (mp. from the literature 194-196 °C);

Elemental analysis calcd for $C_{30}H_{29}N_5O_2$: C 73.30, H 5.95, N 14.25, found: C 73.14, H 6.25, N 14.18.

MS (EI, 70 eV): *m*/*z* = 492.20 $[M]^+$.

^{1}H NMR (400 MHz, DMSO-d_6) δ 7.15 – 8.03 (m, 15H, ArH + NH), 5.64 (s, 1H, CH), 5.25 (d, *J* = 14.3 Hz, 1H, CH_2), 4.57 (d, *J* = 15.0 Hz, 1H, CH_2), 3.14 – 3.26 (m, 1H, cyclohexyl CH), 0.78 – 1.54 (m, 10H, cyclohexyl CH_2).

^{13}C NMR (100 MHz, DMSO-d_6) δ 165.82, 158.91, 148.53, 137.24, 134.30, 131.78, 131.53, 130.44, 130.21, 129.67, 129.14, 128.78, 128.74, 128.72, 128.64, 127.90, 123.24, 63.90, 52.70, 49.04, 32.05, 31.91, 25.40, 25.04.

3. X-ray experimental data

The experimental data were measured on an Xcalibur 3 diffractometer (graphite monochromated MoK_{α} radiation, CCD-detector, ω scanning, $2\Theta_{max}$ = 50° for structures **6aea** and **7aaa** and $2\Theta_{max}$ = 60° for structure **6aaa**). The structures were solved by the direct method using SHELXTL package [2]. Positions of hydrogen atoms were located from electron density difference maps and refined using the riding model with $U_{iso} = nU_{eq}$ (n = 1.5 for methyl groups and 1.2 for other hydrogen atoms). The hydrogen atoms of the amino group were refined isotropically in structure **7aaa**.

The crystals of **6aaa** ($C_{25}H_{24}N_6O_3$) are triclinic. At 293 K, a = 9.6032(11) Å, b = 11.7996(16) Å, c = 11.9647(16) Å, α = 86.08(1)°, β = 80.22(1)°, γ = 70.22(1)°, V = 1257.1(3) Å^3, M_r = 456.50, Z =2, space group $P\bar{1}$,, d_{calc} = 1.206 g/cm^3, $\mu(MoK_{\alpha})$ = 0.082 mm^{-1}, F(000) = 480. Intensities of 12528 reflections (7204 independent, R_{int} = 0.082) were measured. Full-matrix least-squares refinement against F^2 in anisotropic approximation for non-hydrogen atoms was converged to wR_2 = 0.189 for 7204 reflections (R_1 = 0.069 for 1731 reflections with $F > 4\sigma(F)$, S = 0.750).

The crystals of **6aea** $C_{28}H_{27}N_5O_2$, are orthorhombic. At 293 K, a = 31.807(2) Å, b = 11.8007(6) Å, c = 6.7991(6) Å, V = 2552.0(3) Å^3, M_r = 465.54, Z = 4, space group $Pna2_1$, d_{calc} = 1.212 g/cm^3, $\mu(MoK_{\alpha})$ = 0.079 mm^{-1}, F(000) = 984. Intensities of 17590 reflections (4461 independent, R_{int}=0.114) were measured. Full-matrix least-squares refinement against F^2 in anisotropic approximation for non-hydrogen atoms was converged to wR_2 = 0.236 for 4461 reflections (R_1 = 0.080 for 2952 reflections with $F > 4\sigma(F)$, S = 0.991).

The crystals of **7aaa** $C_{25}H_{24}N_6O_3$ are monoclinic. At 293 K, a = 10.9652(13) Å, b = 14.0758(15) Å, c = 14.8615(10) Å, β = 93.086(6)°, V = 2290.5(4) Å^3, M_r = 456.50, Z =4, space group $P2_1/c$, d_{calc} = 1.324 g/cm^3, $\mu(MoK_{\alpha})$ = 0.090 mm^{-1}, F(000) = 960. Intensities of 16271 reflections (3975 independent, R_{int}=0.115) were measured. Full-matrix least-squares refinement against F^2 in anisotropic approximation for non-hydrogen atoms was converged to wR_2 = 0.183 for 3975 reflections (R_1 = 0.065 for 2014 reflections with $F > 4\sigma(F)$, S = 0.911).

Final atomic coordinates, geometrical parameters, and crystallographic data have been deposited with the Cambridge Crystallographic Data Centre, 11 Union Road, Cambridge, CB2 1EZ, UK (E-mail: deposit@ccdc.cam.ac.uk; fax: +44 1223 336033) and are available on request quoting the deposition numbers 2042893 (**6aaa**), 2042895 (**6aea**) and 2042894 (**7aaa**).

Literature

(1) Vachhani, D. D.; Kumar, A.; Modha, S. G.; Sharma, S. K.; Parmar, V. S.; Van der Eycken, E. V. *Eur. J. Org. Chem* **2013**, 1223–1227

(2) Sheldrick, G. M. A Short History of SHELX. *Acta Crystallogr. Sect. A Found. Crystallogr.* **2008**, *64*, 112–122

4. ^{1}H and ^{13}C NMR spectra

^{1}H NMR spectra of **6aaa**

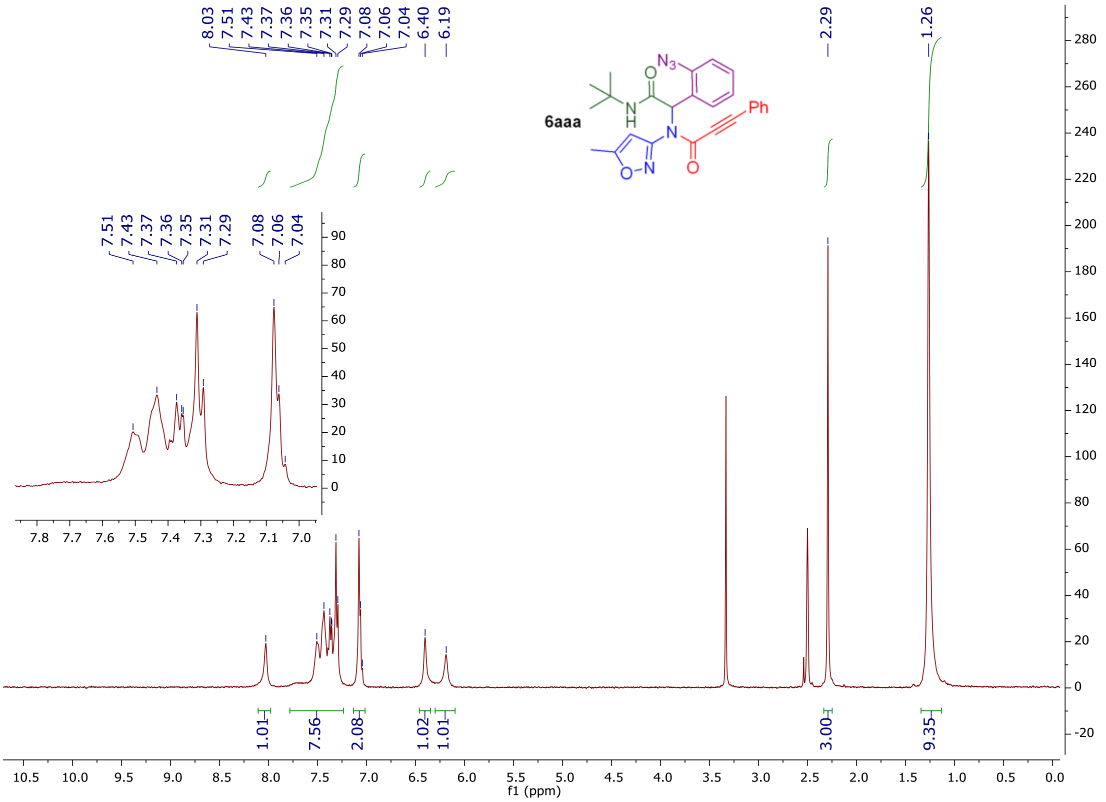

^{1}H NMR spectra of **6aba**

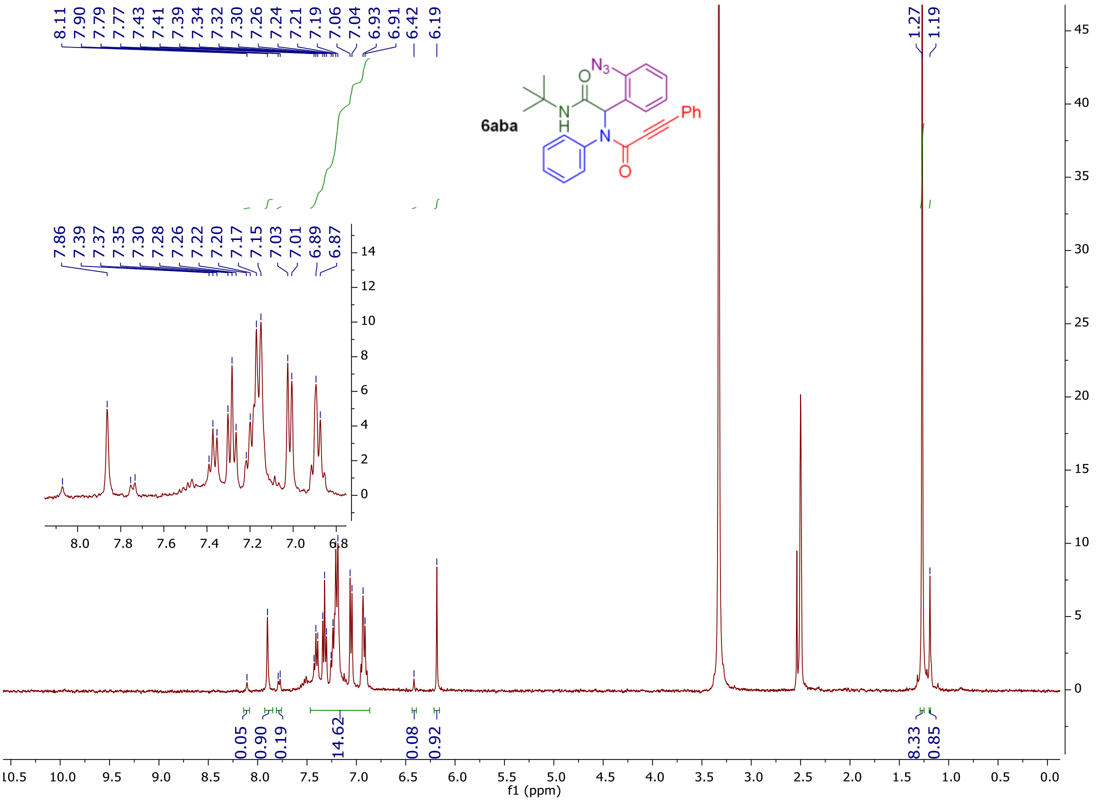

^{1}H NMR spectra of **6aca**

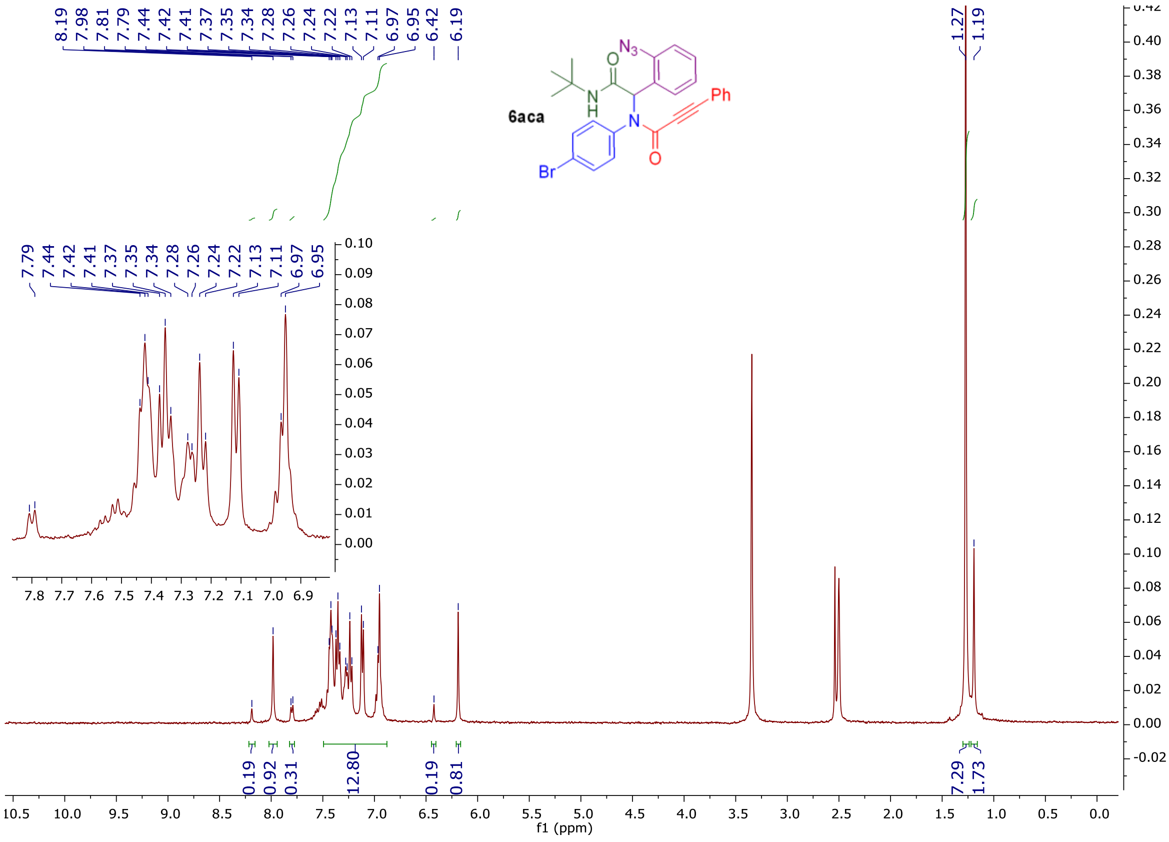

^{1}H NMR spectra of **6ada**

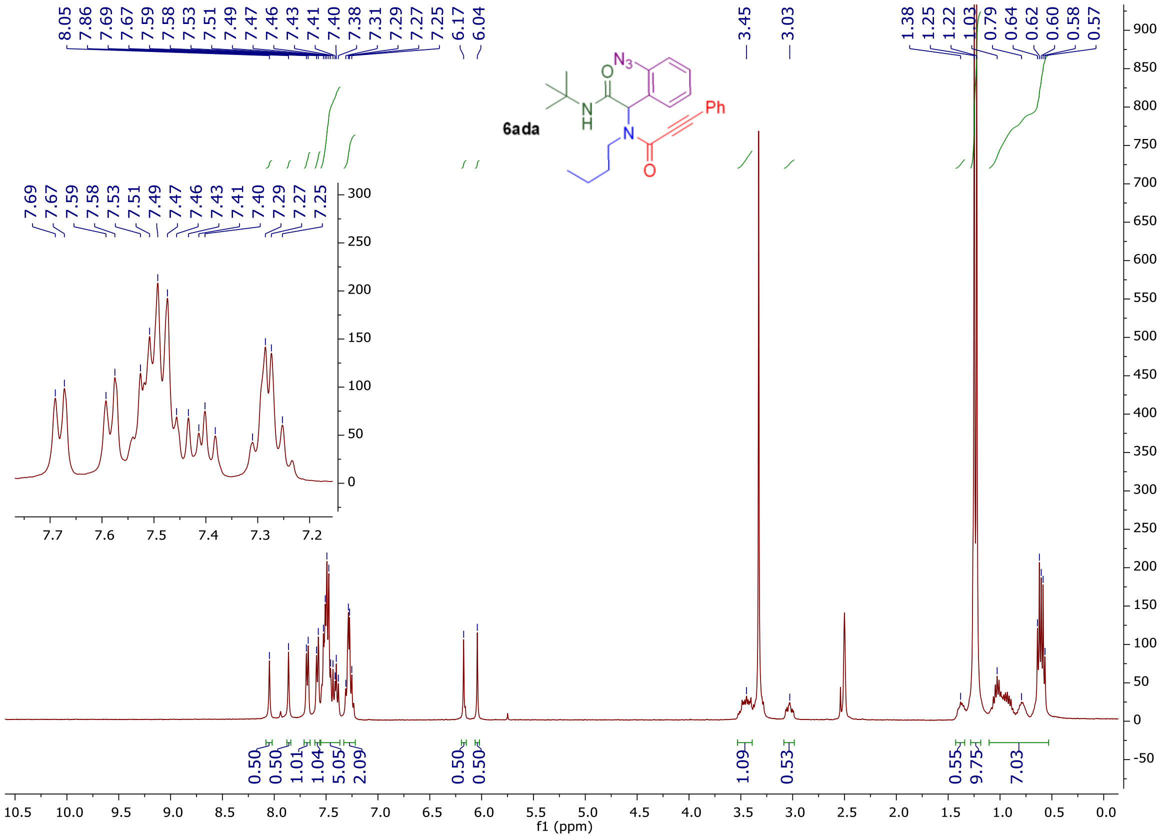

^{1}H NMR spectra of **6aea**

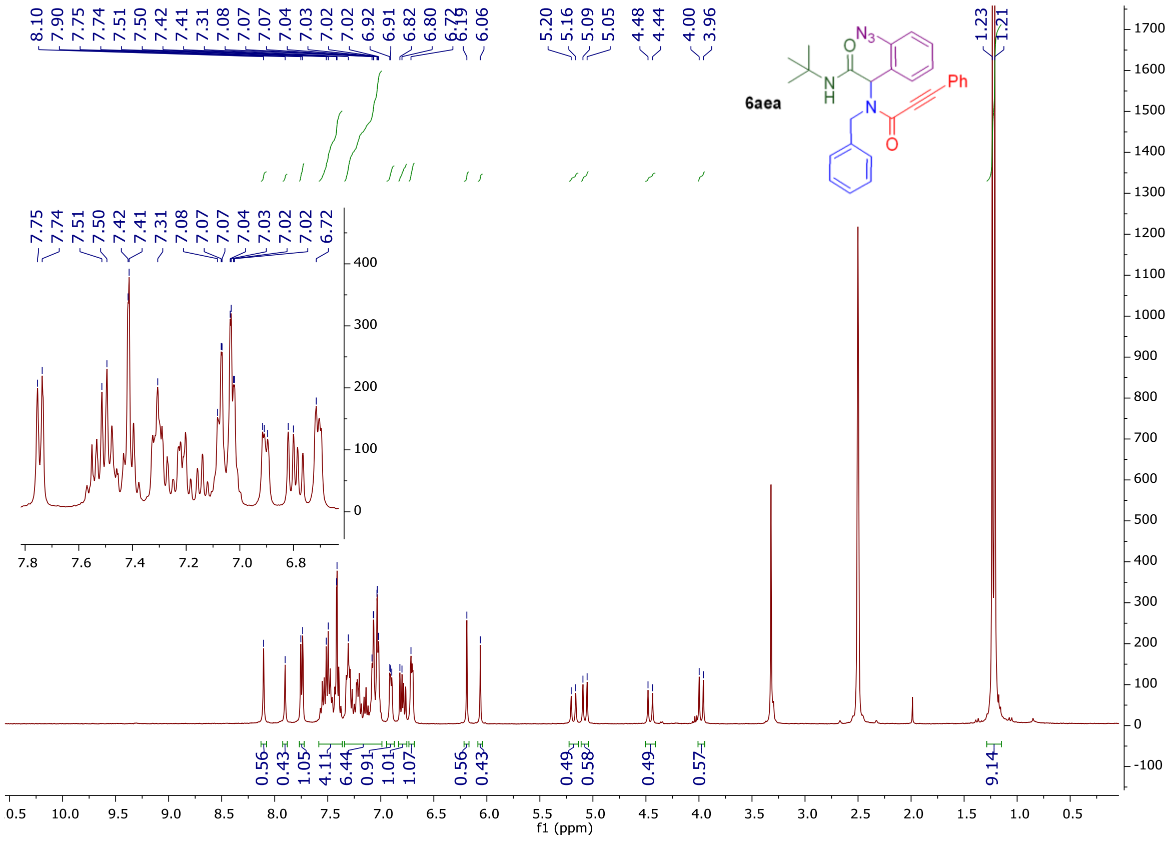

^{1}H NMR spectra of **6aab**

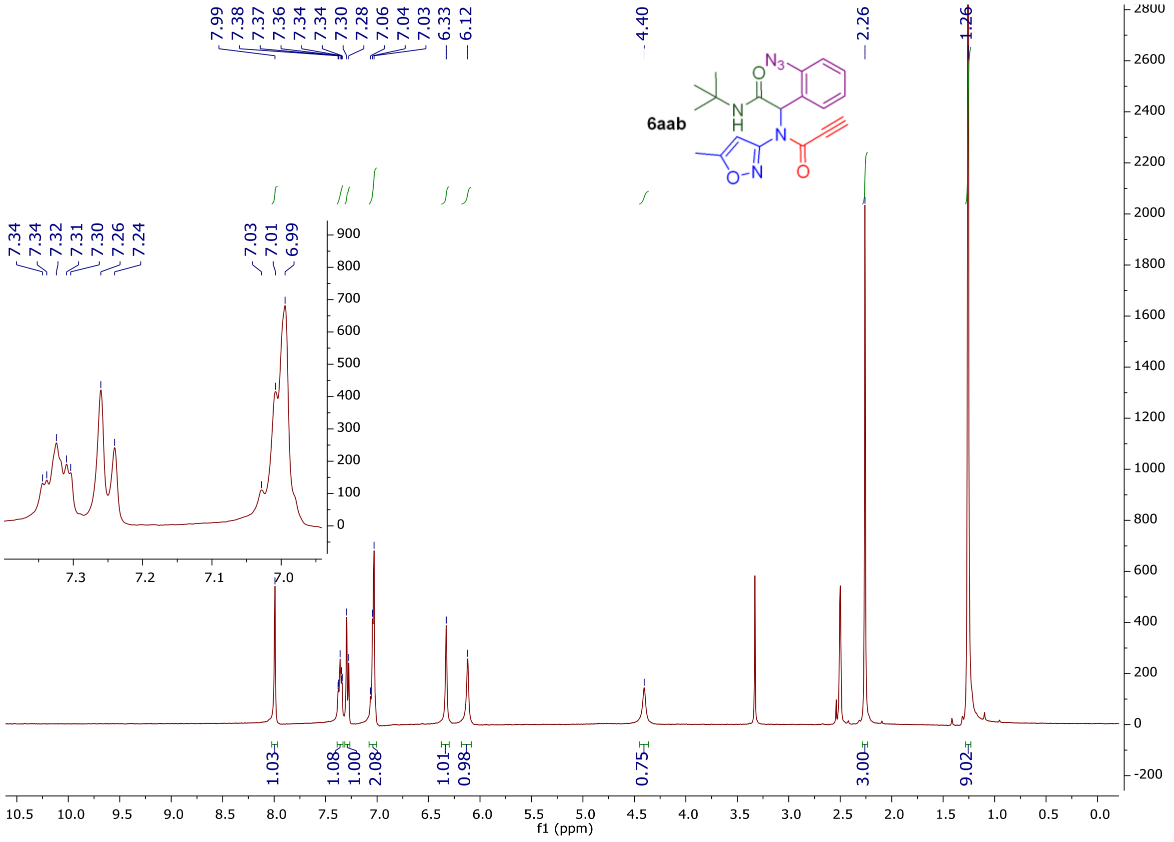

^{1}H NMR spectra of **6abb**

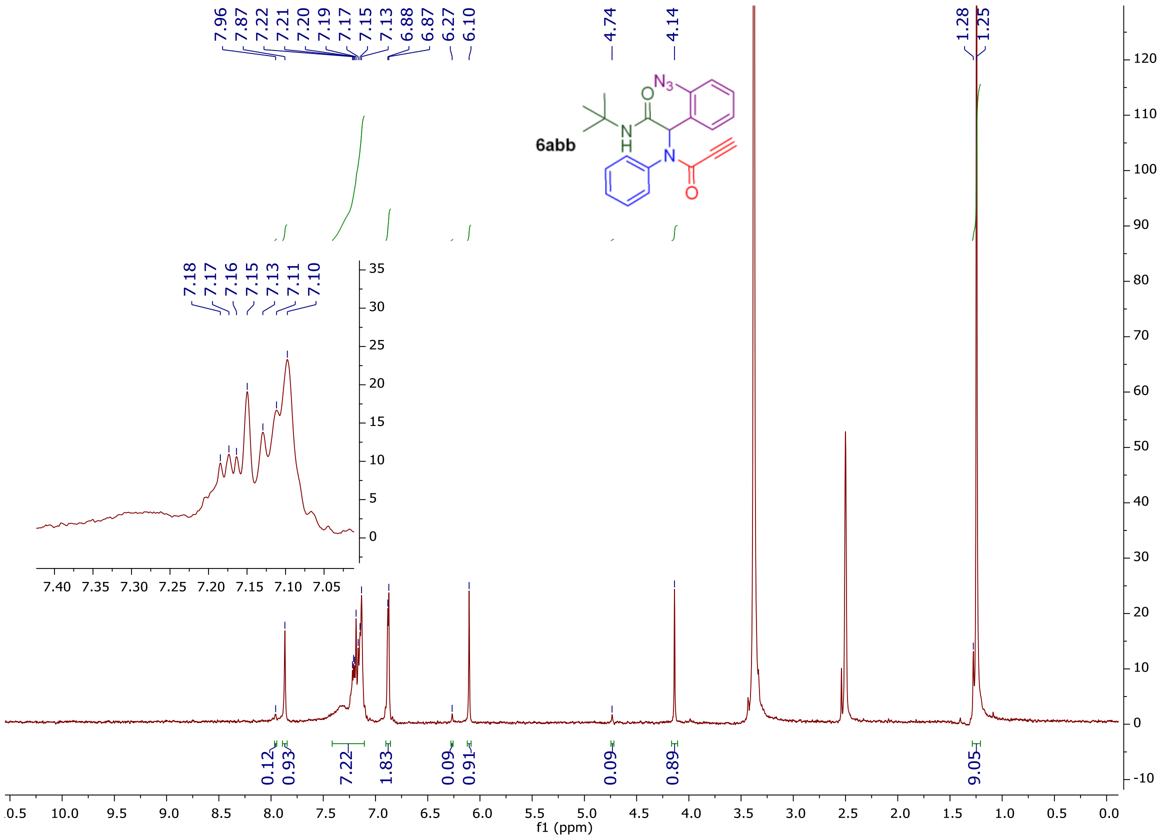

^{1}H NMR spectra of **6aeb**

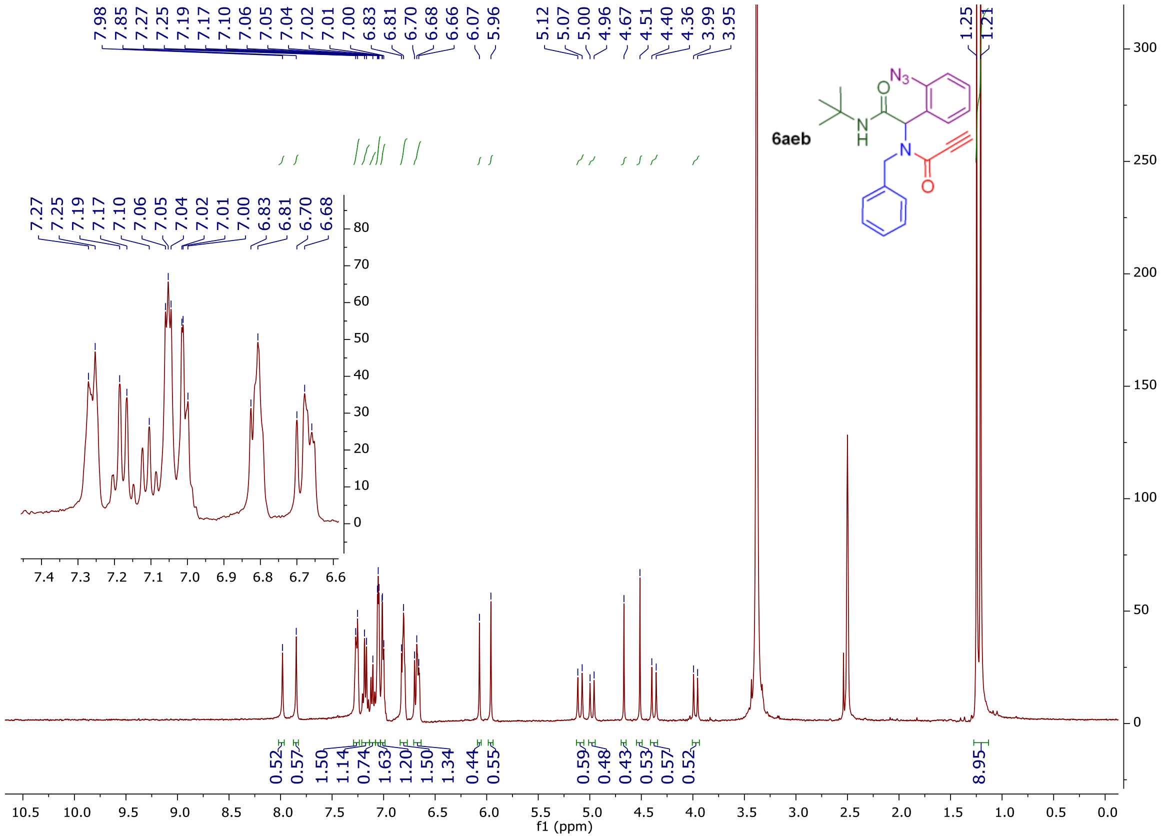

^{1}H NMR spectra of **6baa**

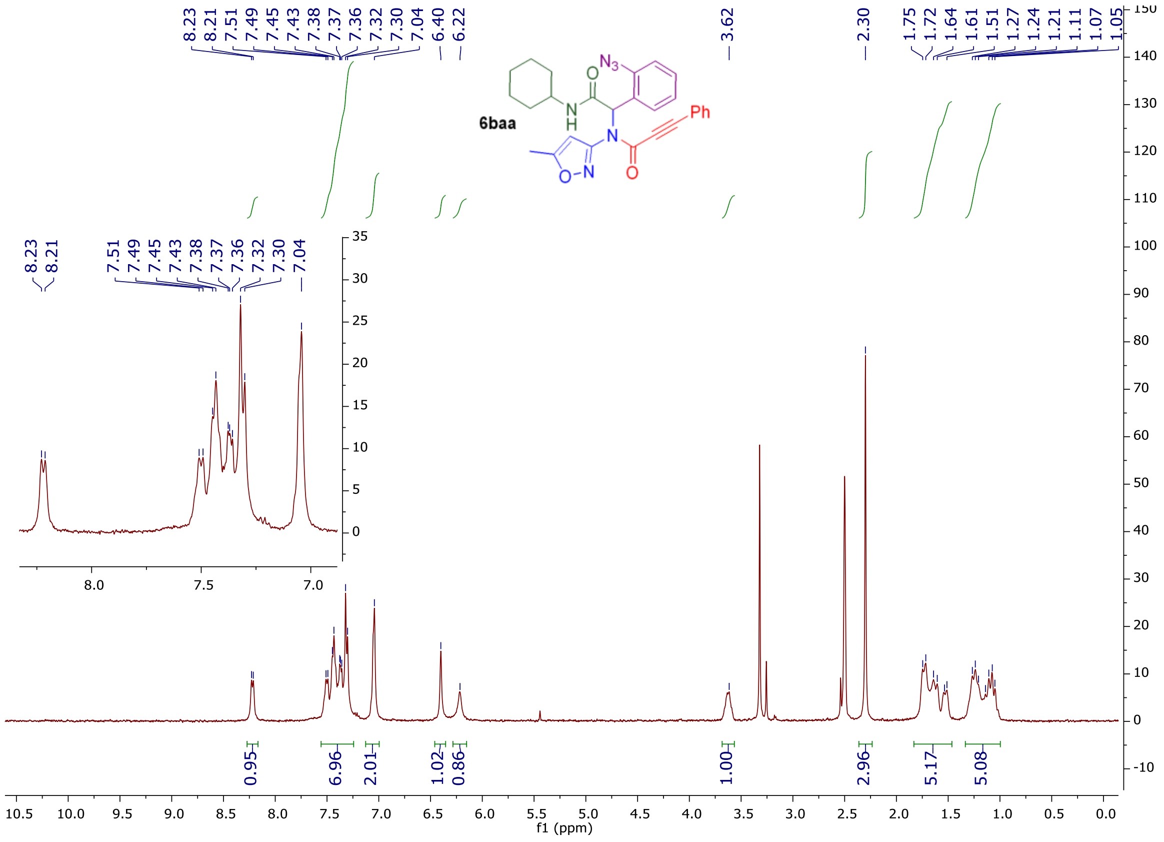

^{1}H NMR spectra of **6bba**

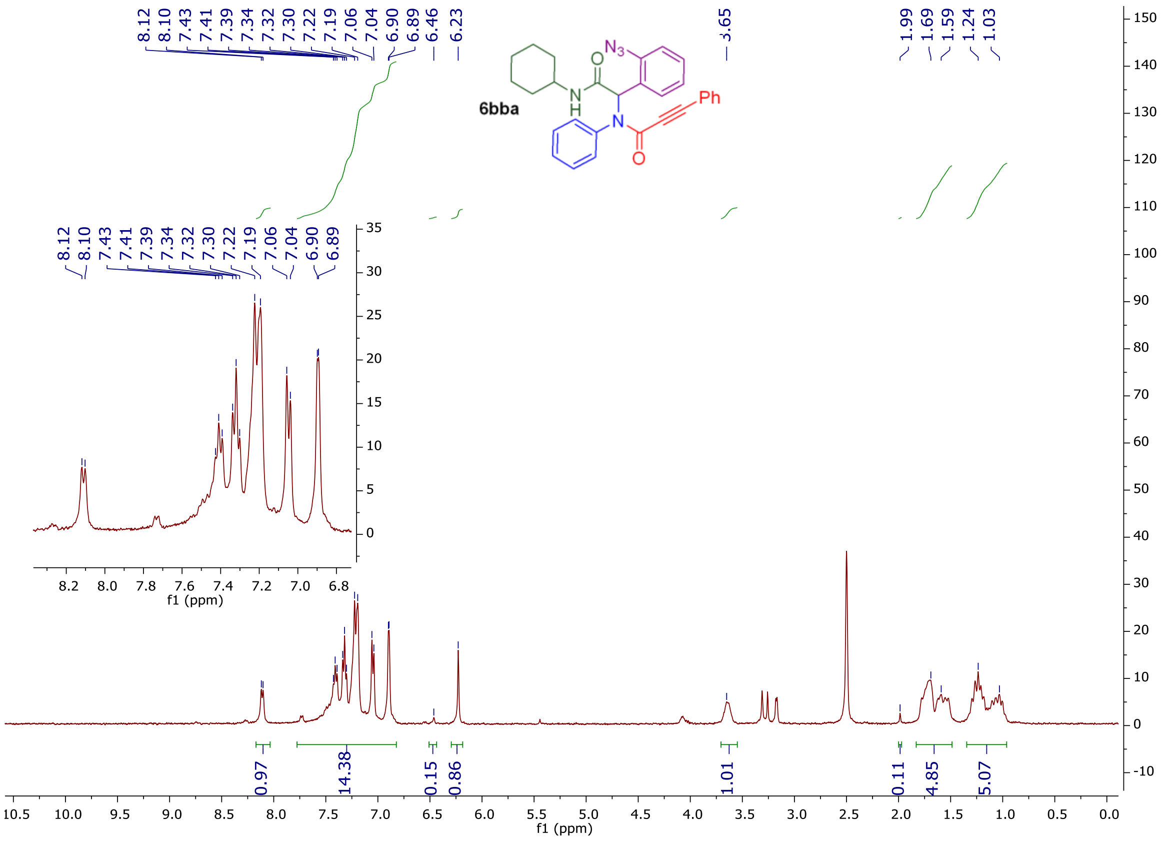

^{1}H NMR spectra of **6bea**

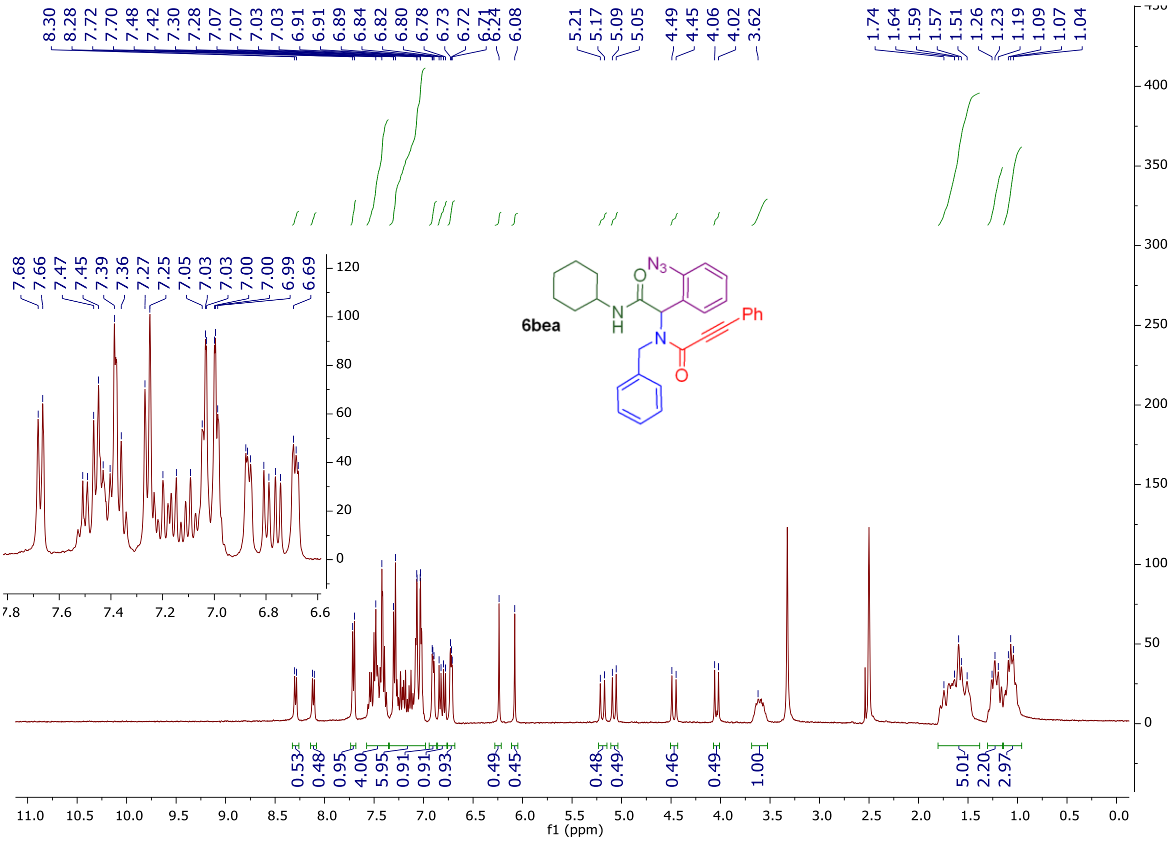

^{1}H NMR spectra of **7aaa**

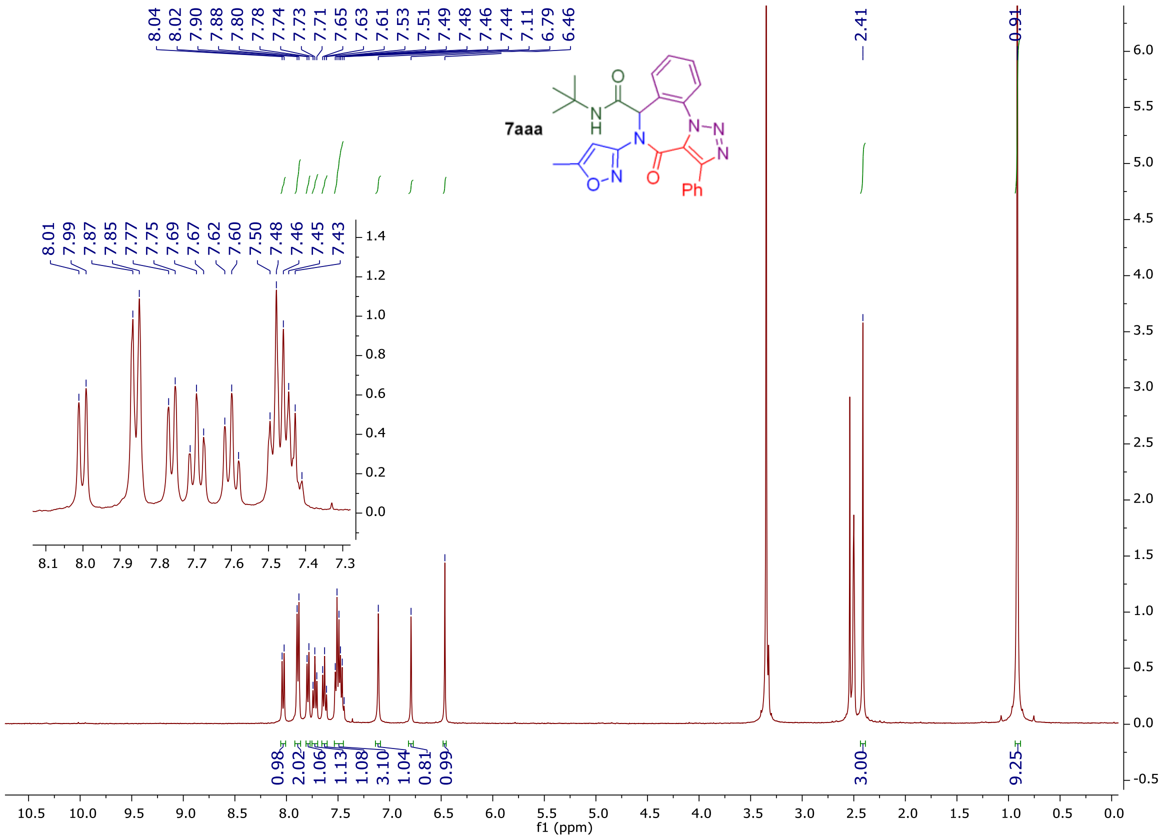

^{1}H NMR spectra of **7aba**

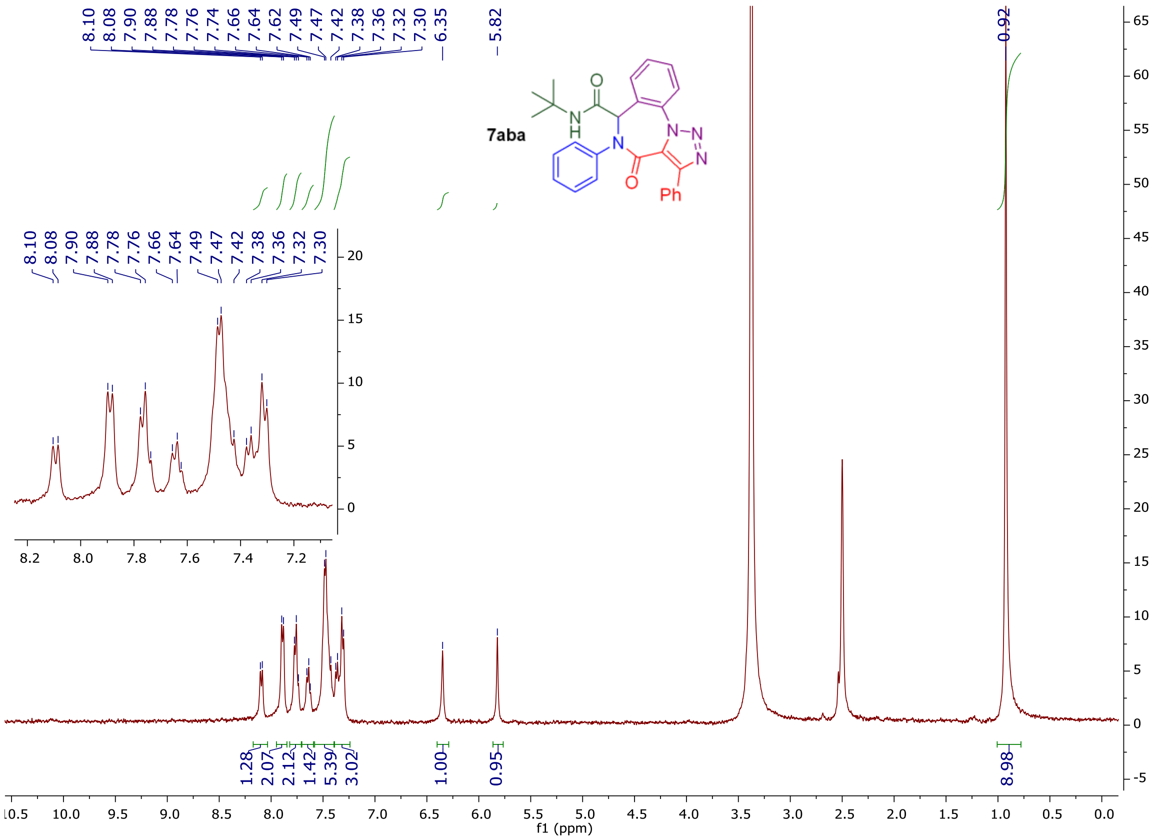

^{1}H NMR spectra of **7aca**

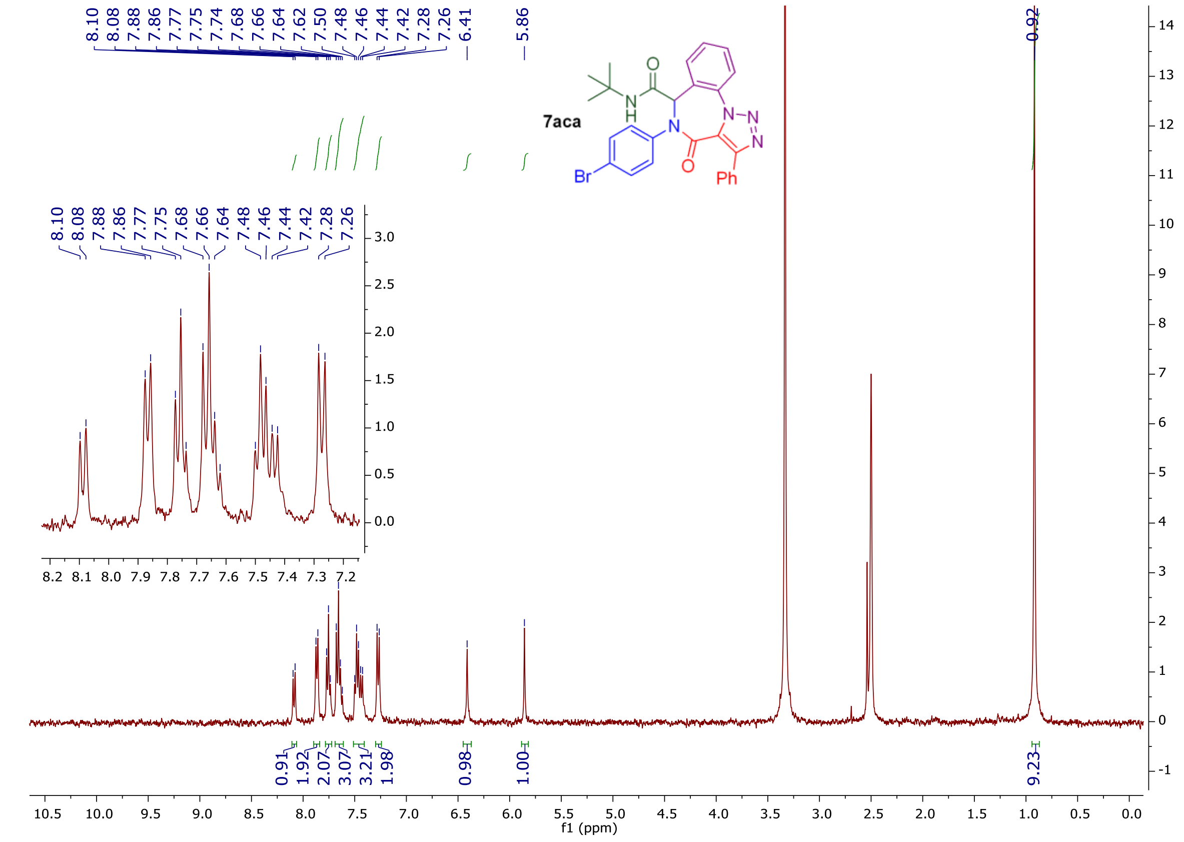

^{1}H NMR spectra of **7ada**

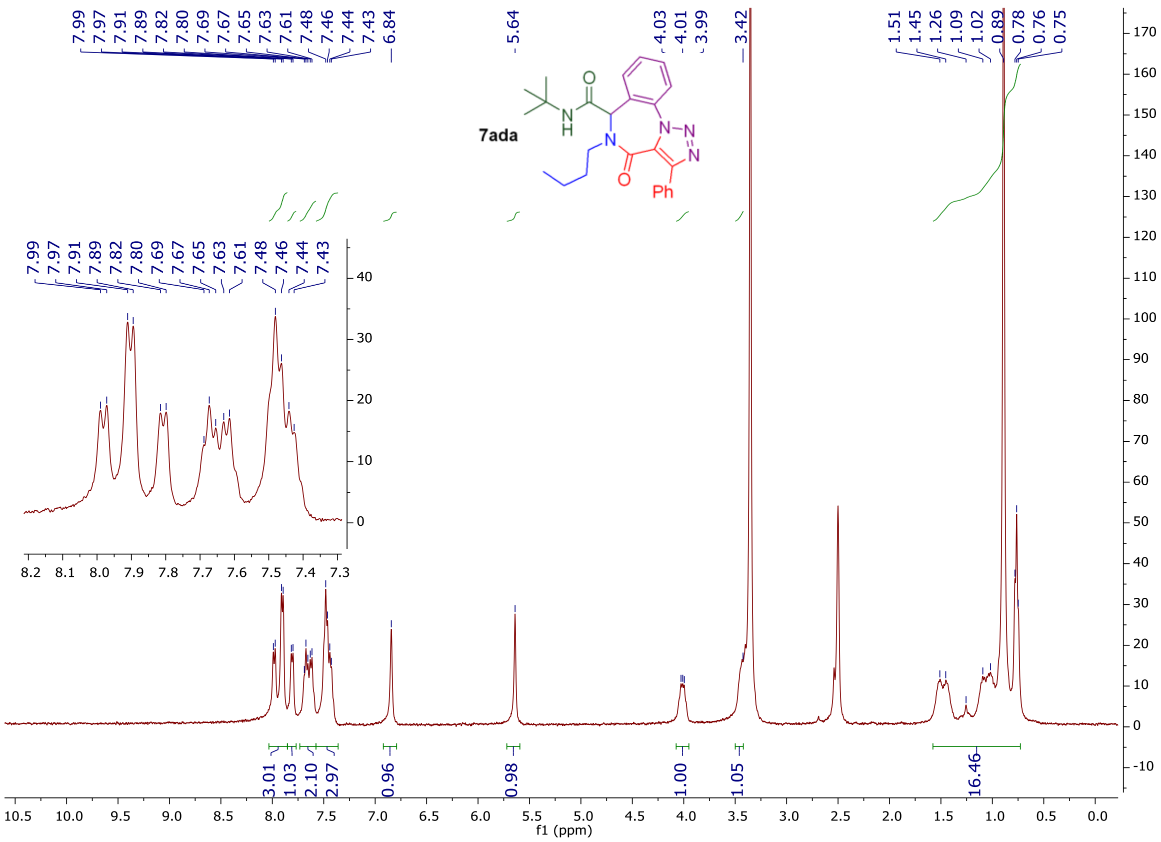

^{1}H NMR spectra of **7aea**

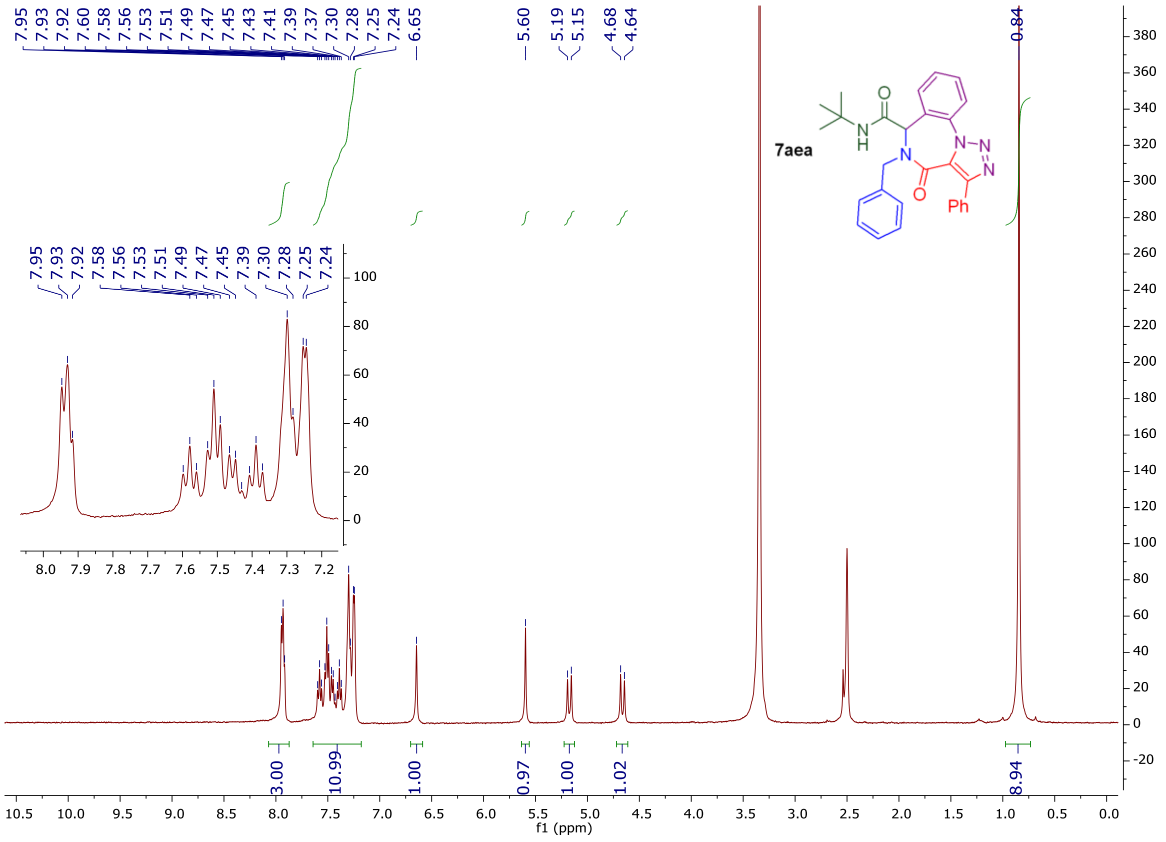

^{1}H NMR spectra of **7aab**

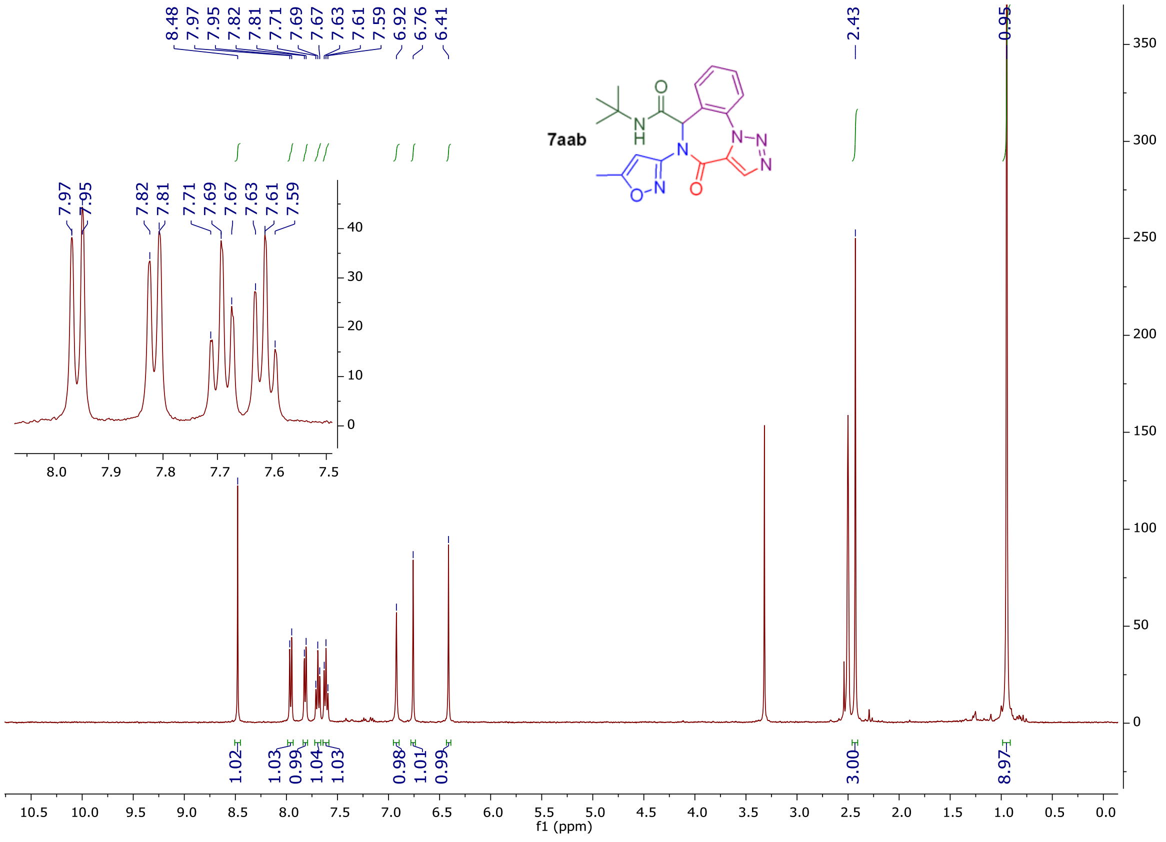

^{1}H NMR spectra of **7abb**

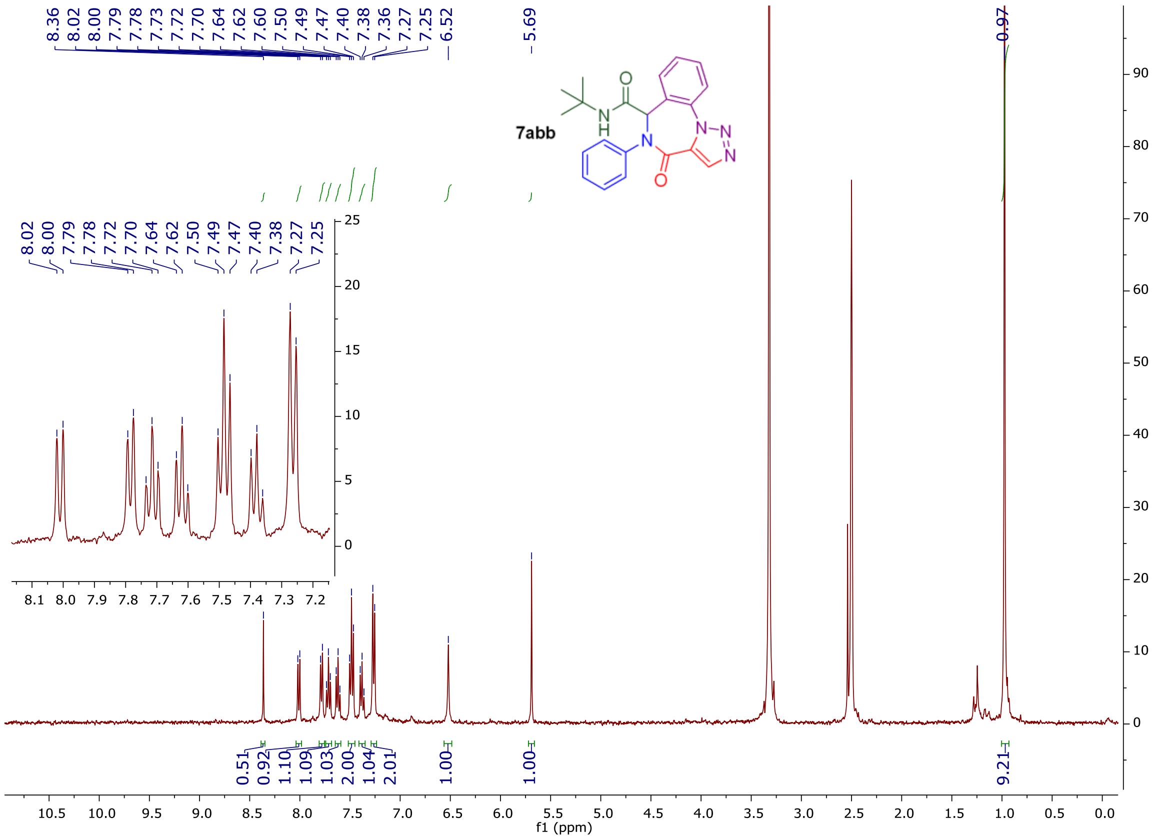

^{1}H NMR spectra of **7aeb**

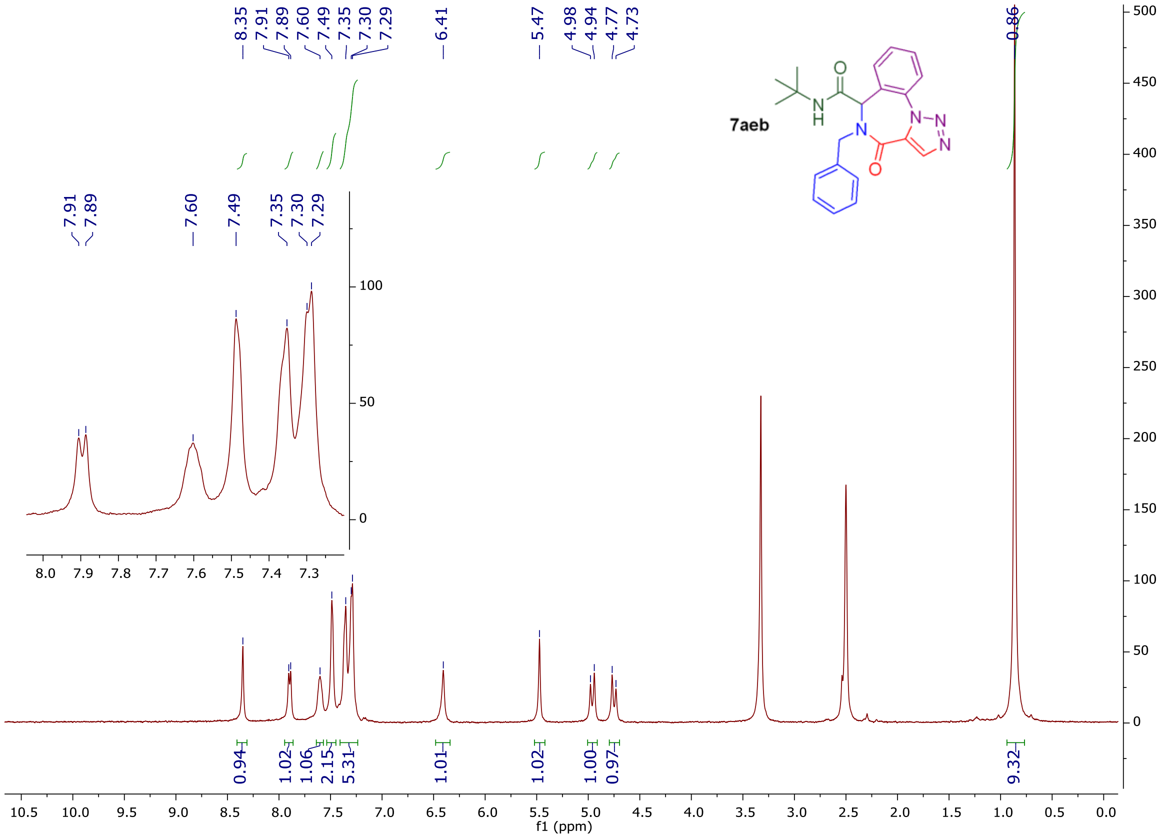

^{1}H NMR spectra of **7baa**

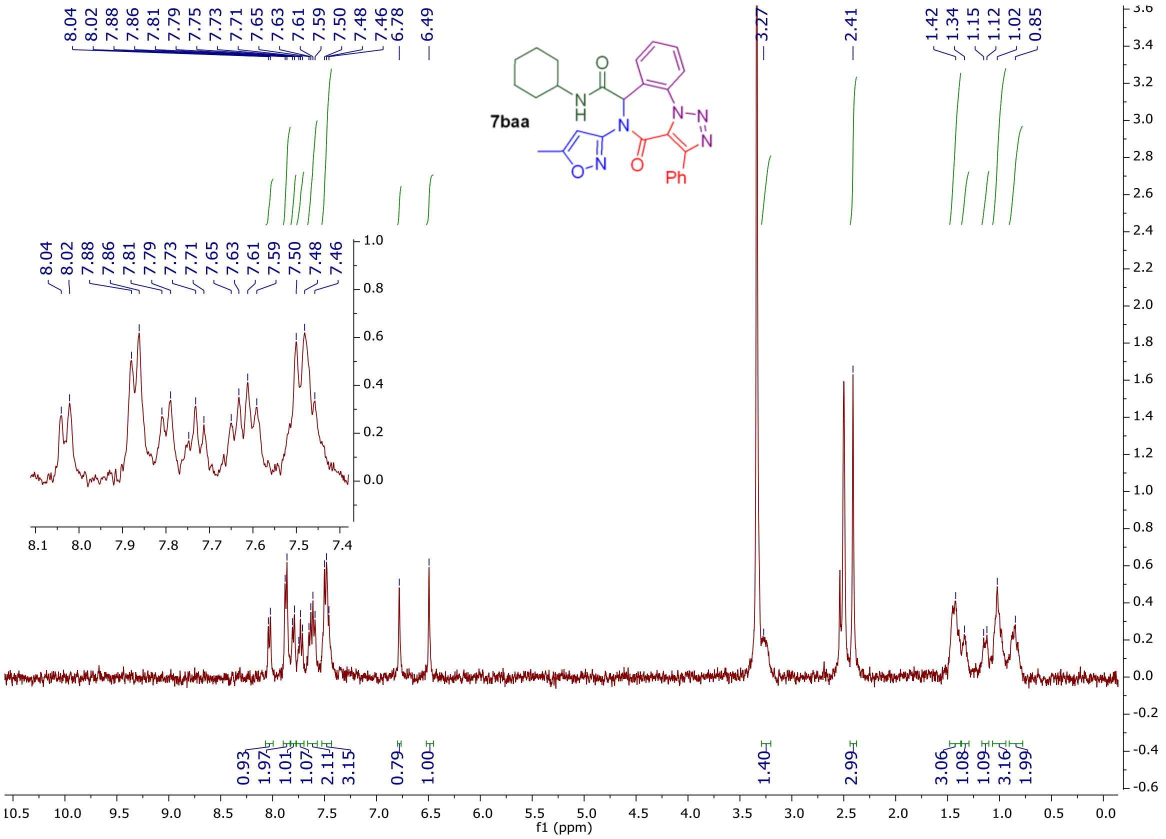

^{1}H NMR spectra of **7bba**

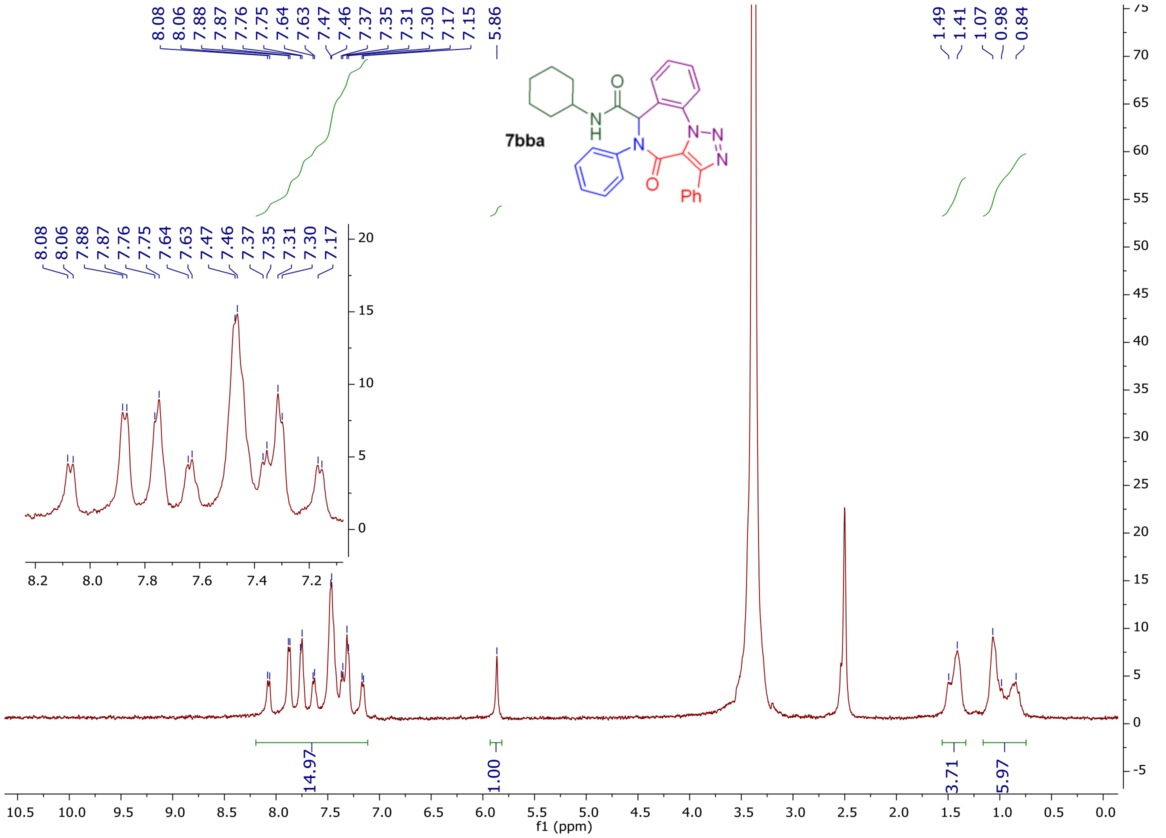

^{1}H NMR spectra of **7bea**

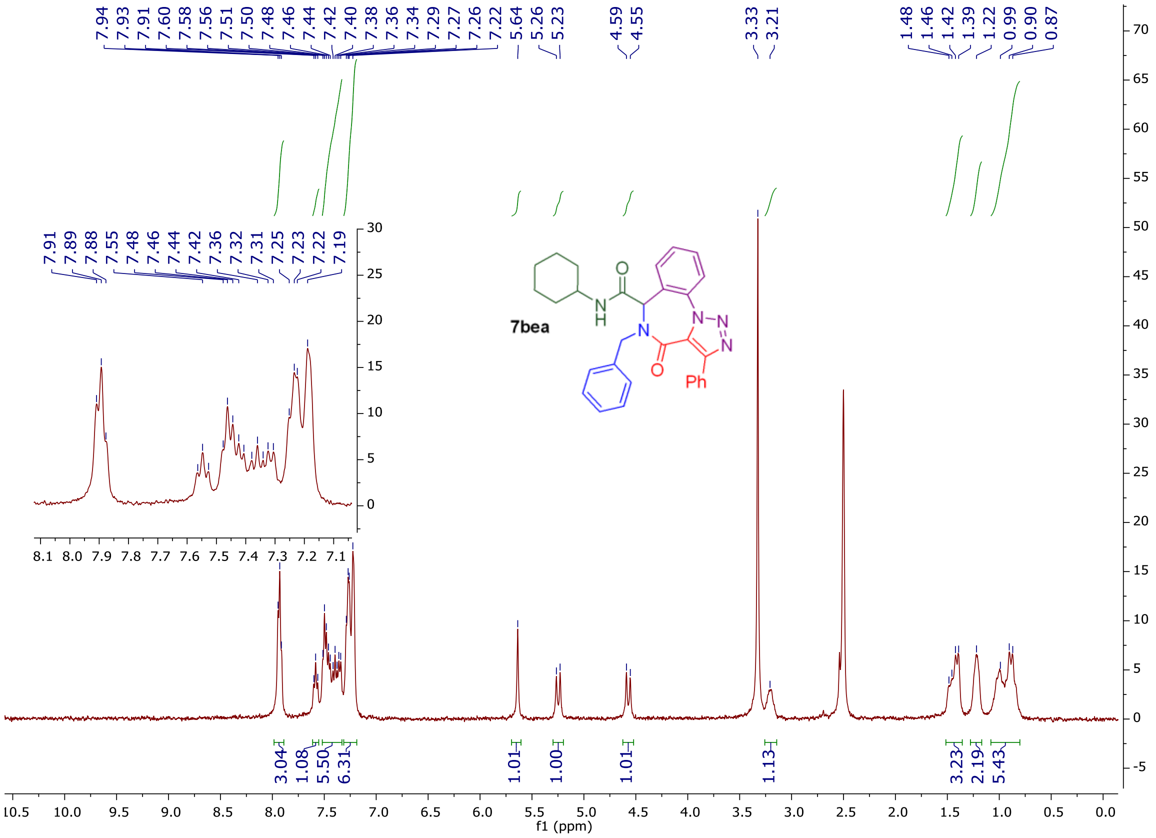

^{13}C NMR spectra of **6aaa**

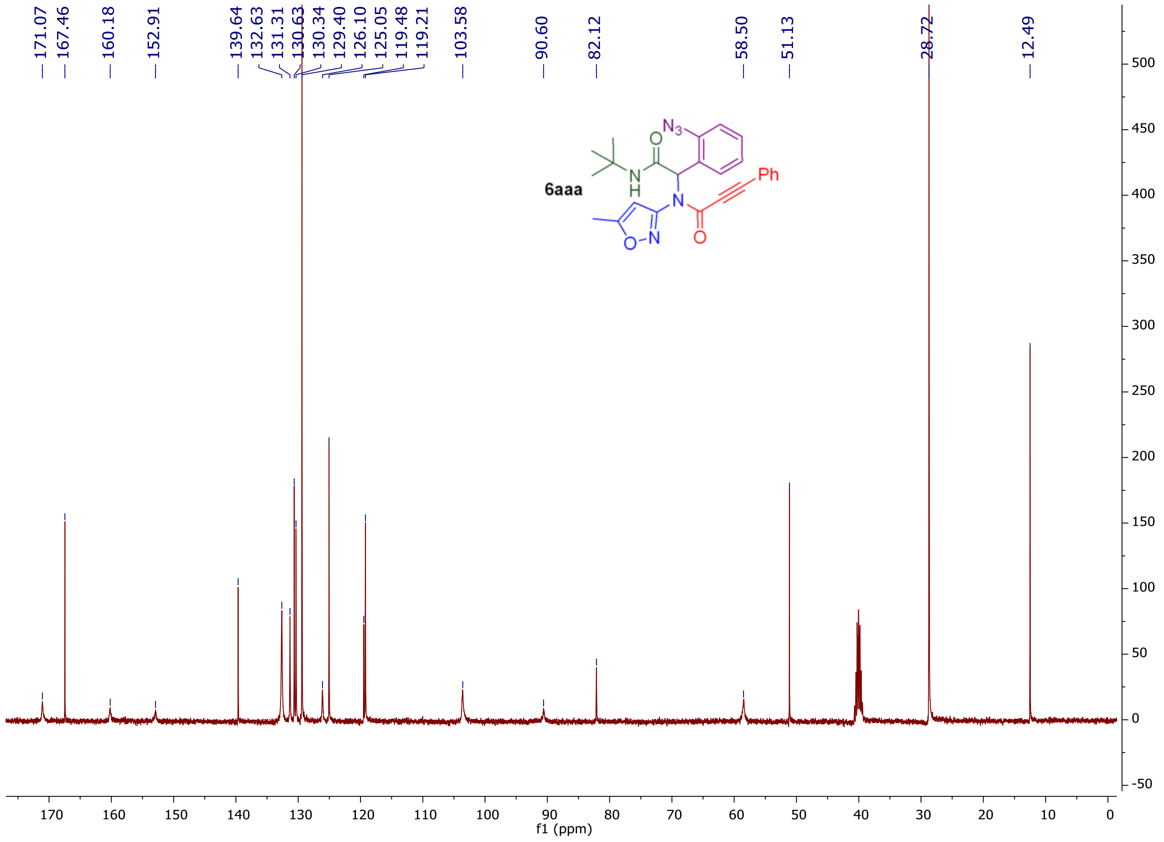

^{13}C NMR spectra of **6aba**

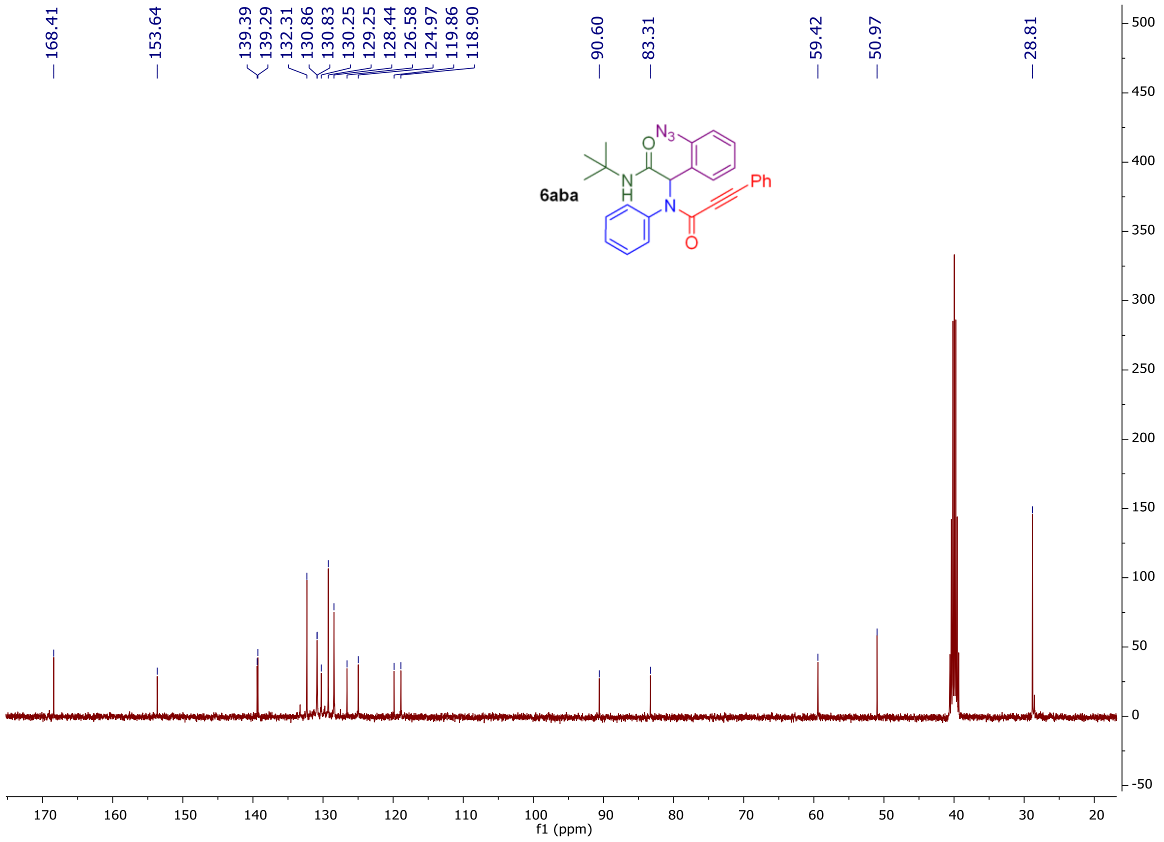

^{13}C NMR spectra of **6aca**

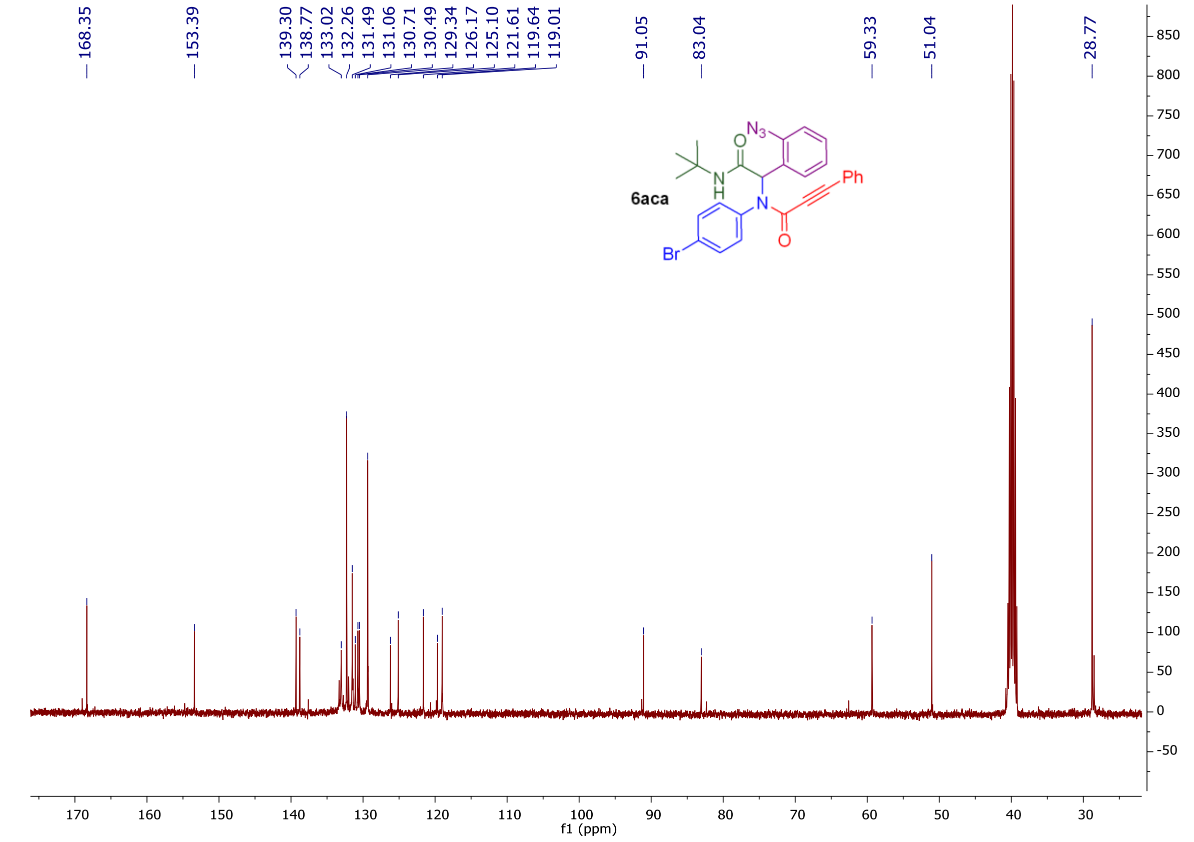

^{13}C NMR spectra of **6ada**

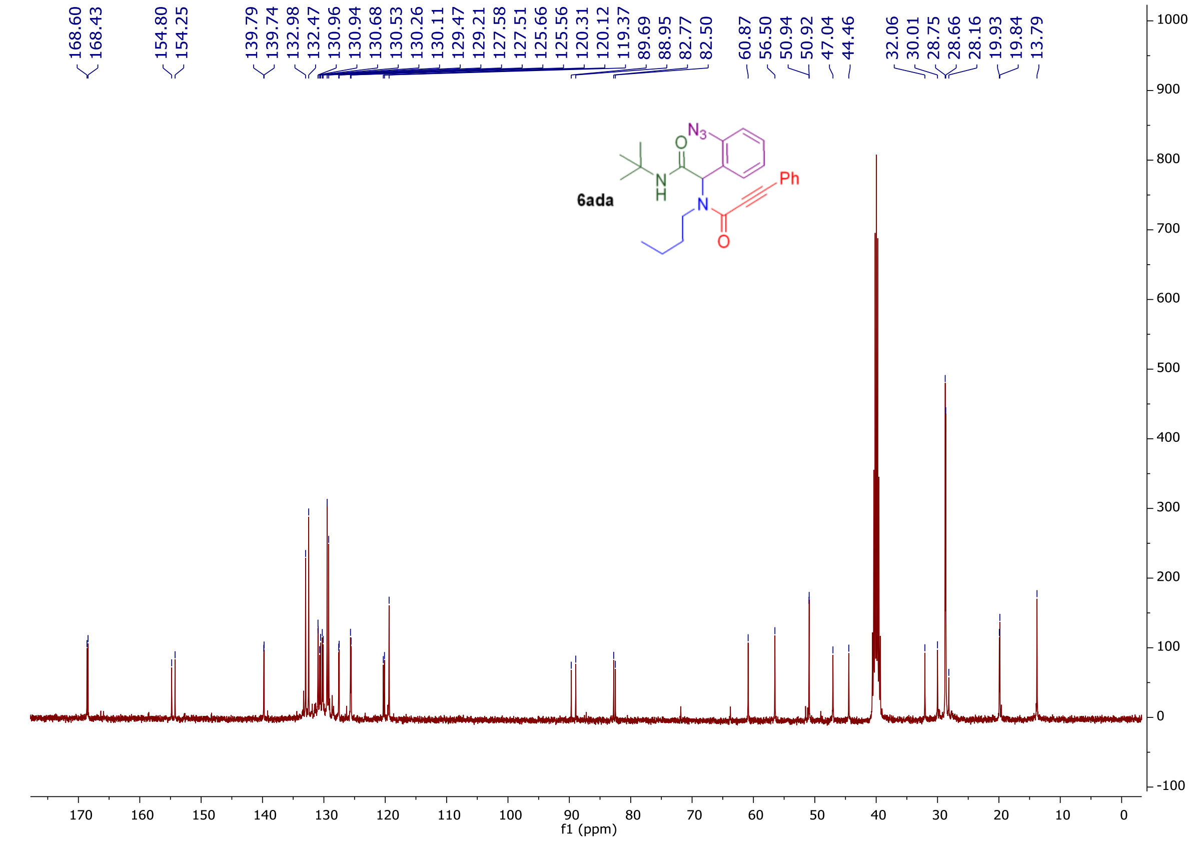

^{13}C NMR spectra of **6aea**

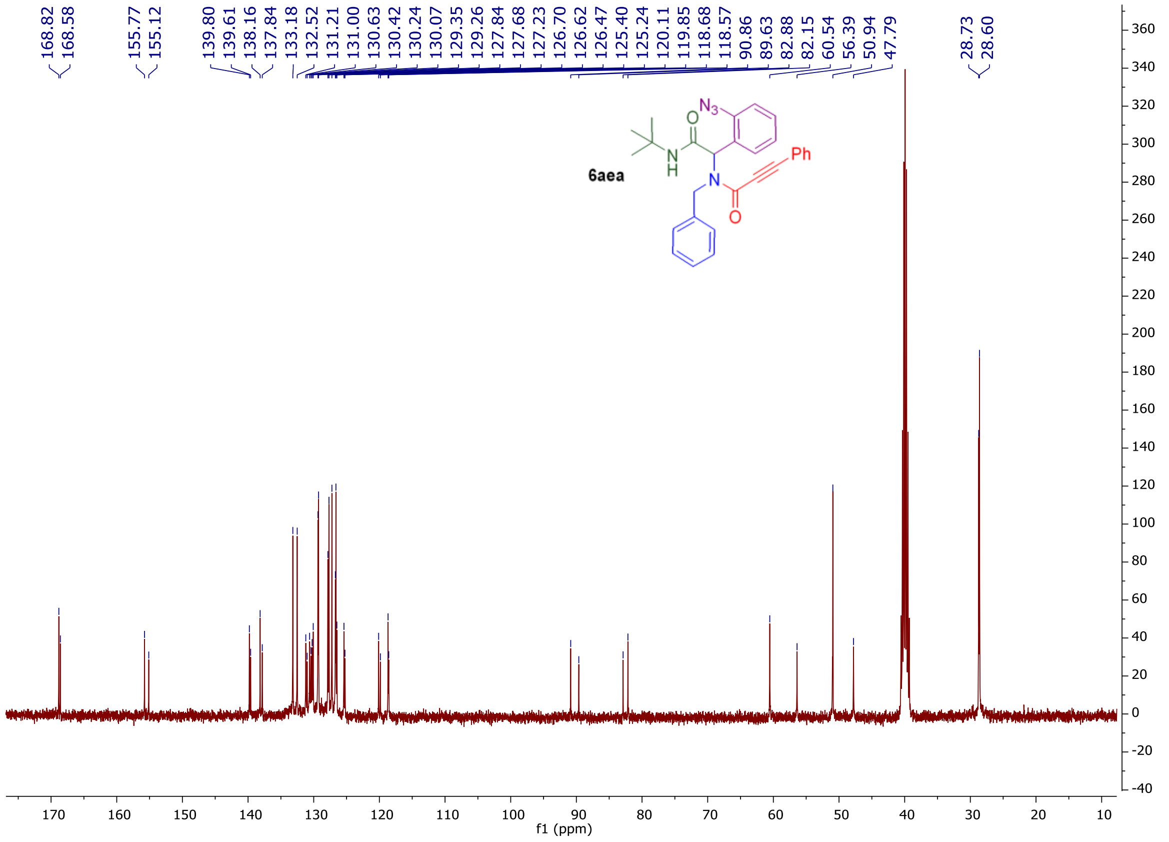

^{13}C NMR spectra of **6aab**

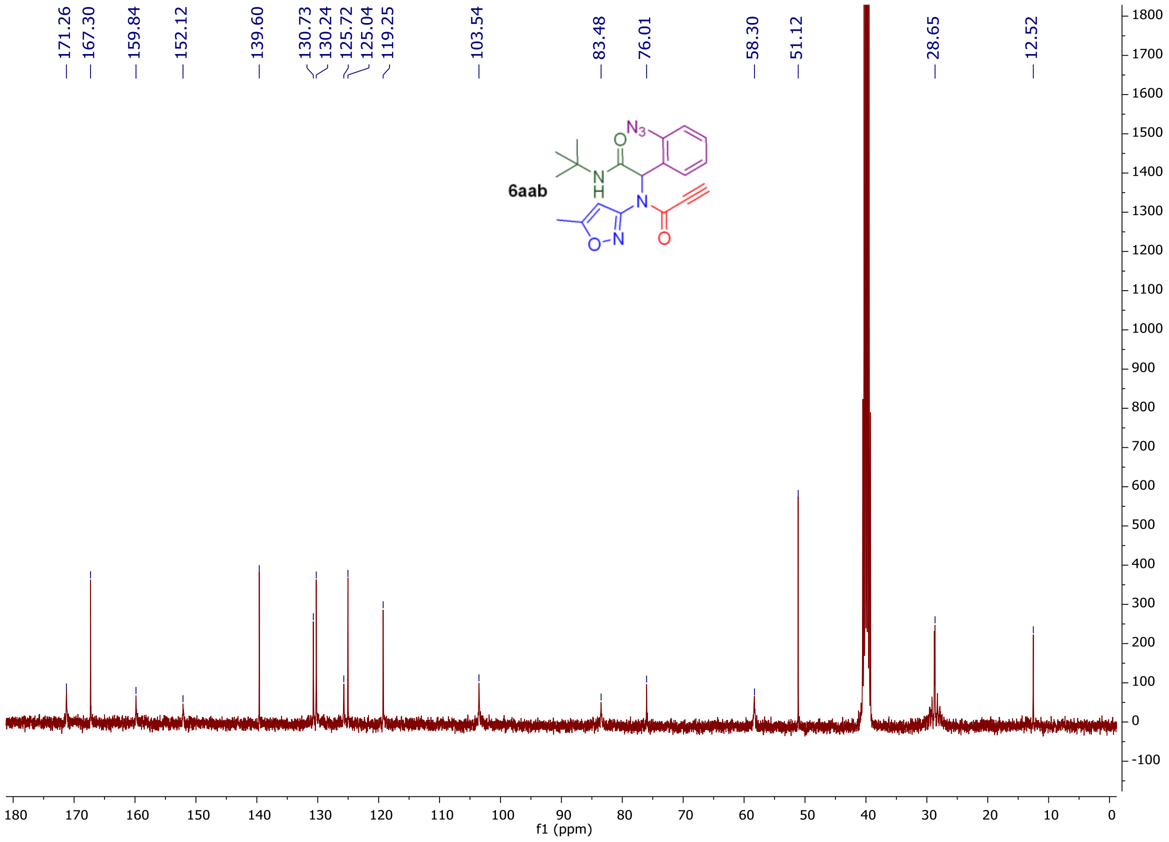

^{13}C NMR spectra of **6abb**

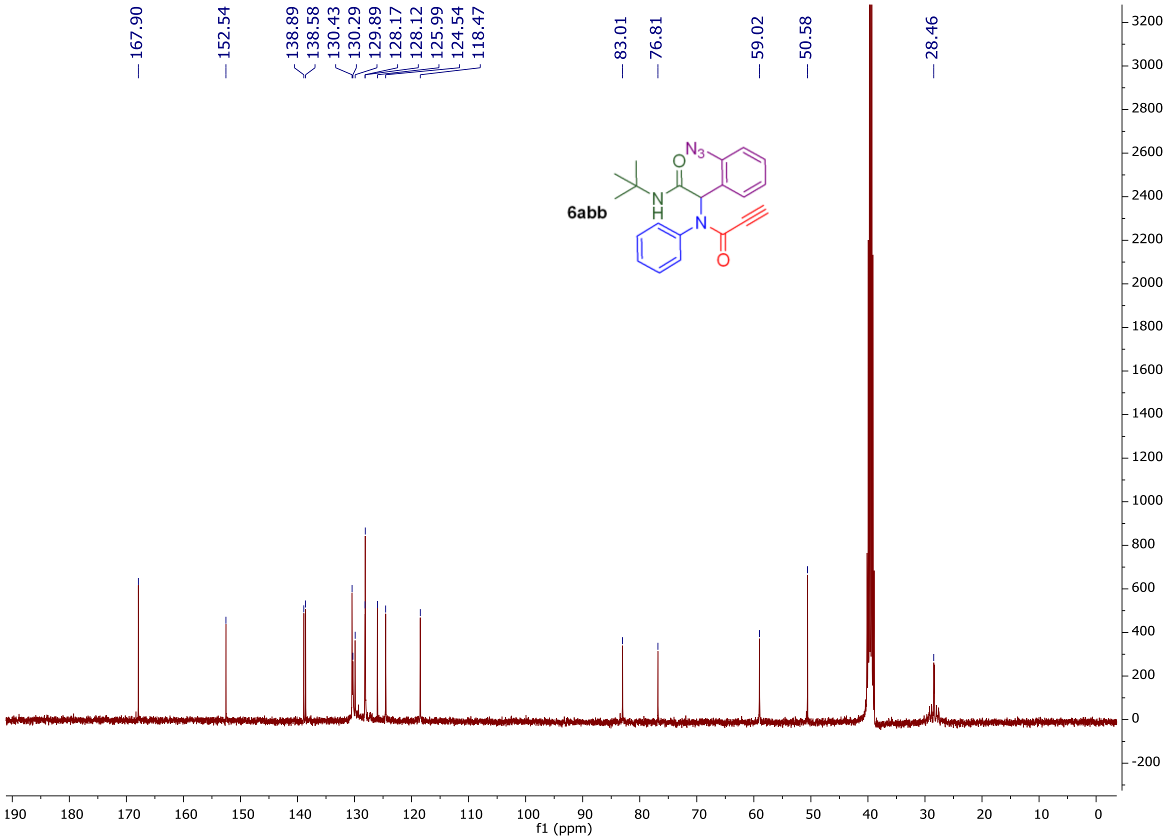

^{13}C NMR spectra of **6aeb**

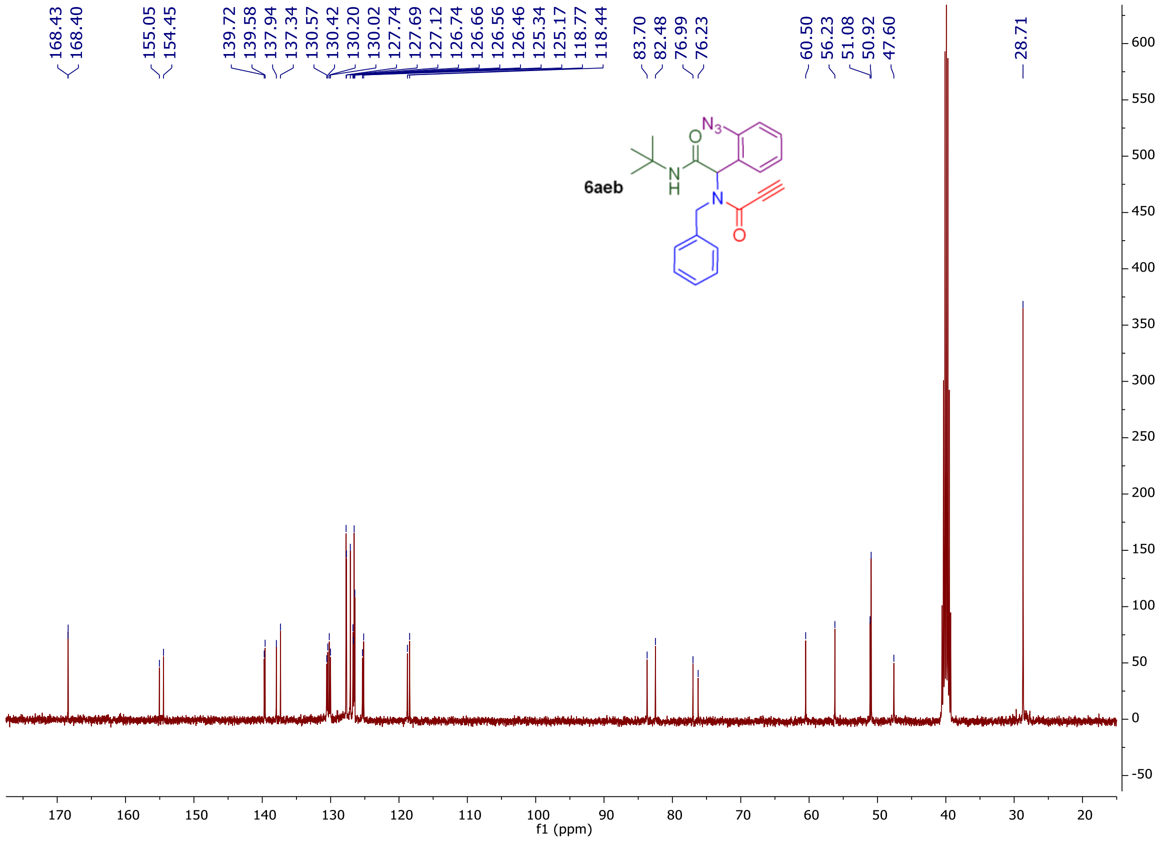

^{13}C NMR spectra of **6baa**

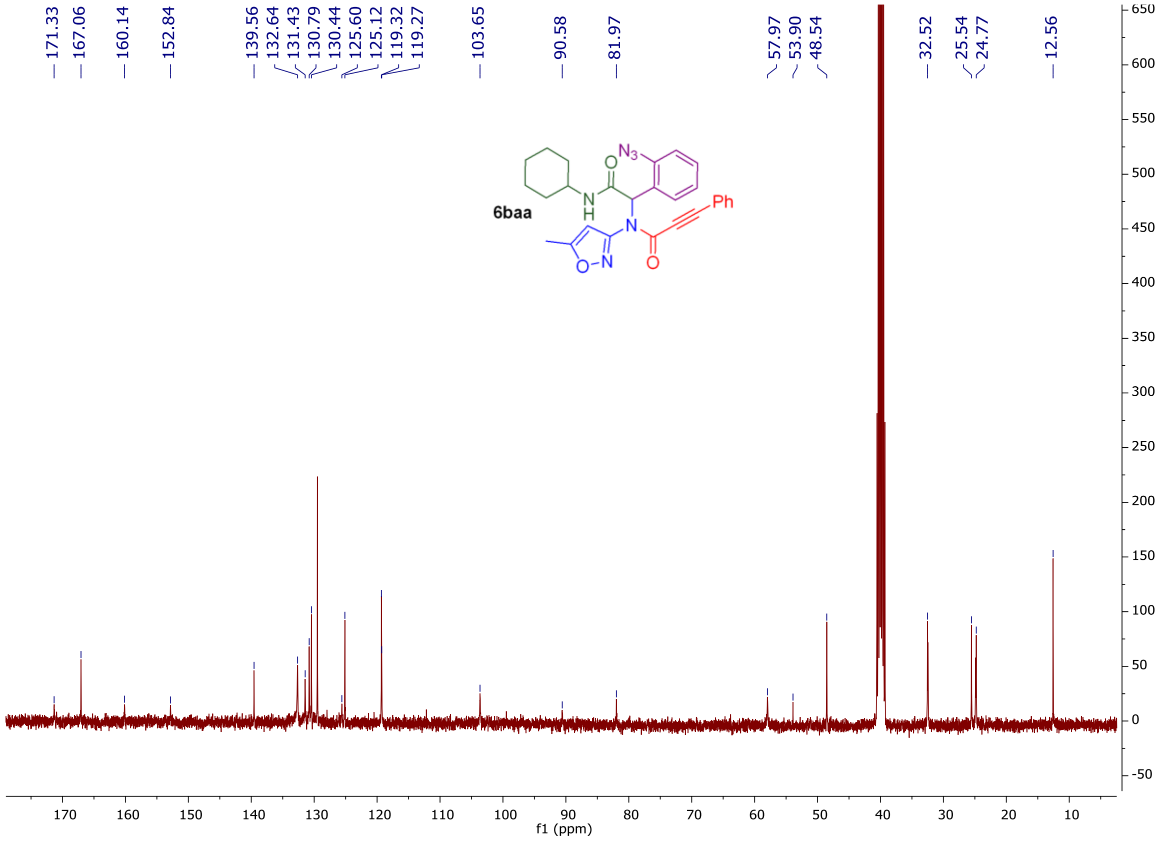

^{13}C NMR spectra of **6bba**

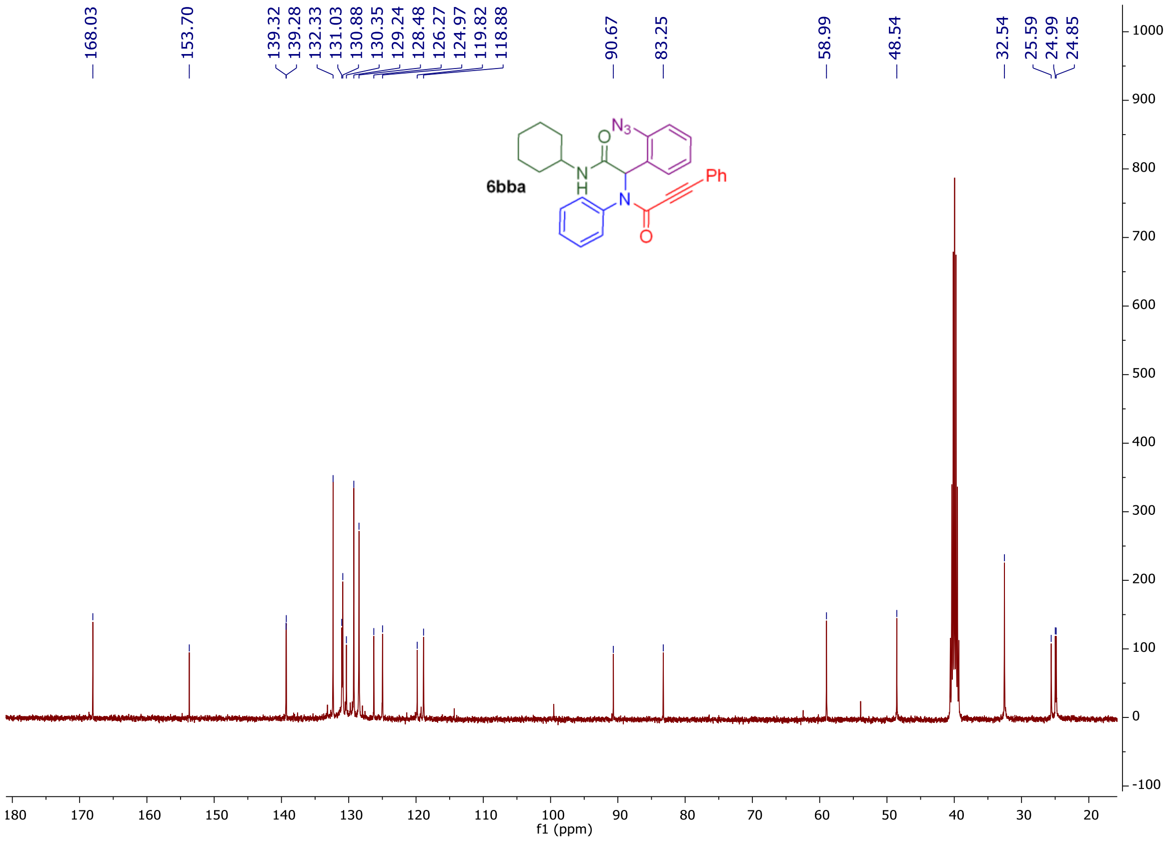

^{13}C NMR spectra of **6bea**

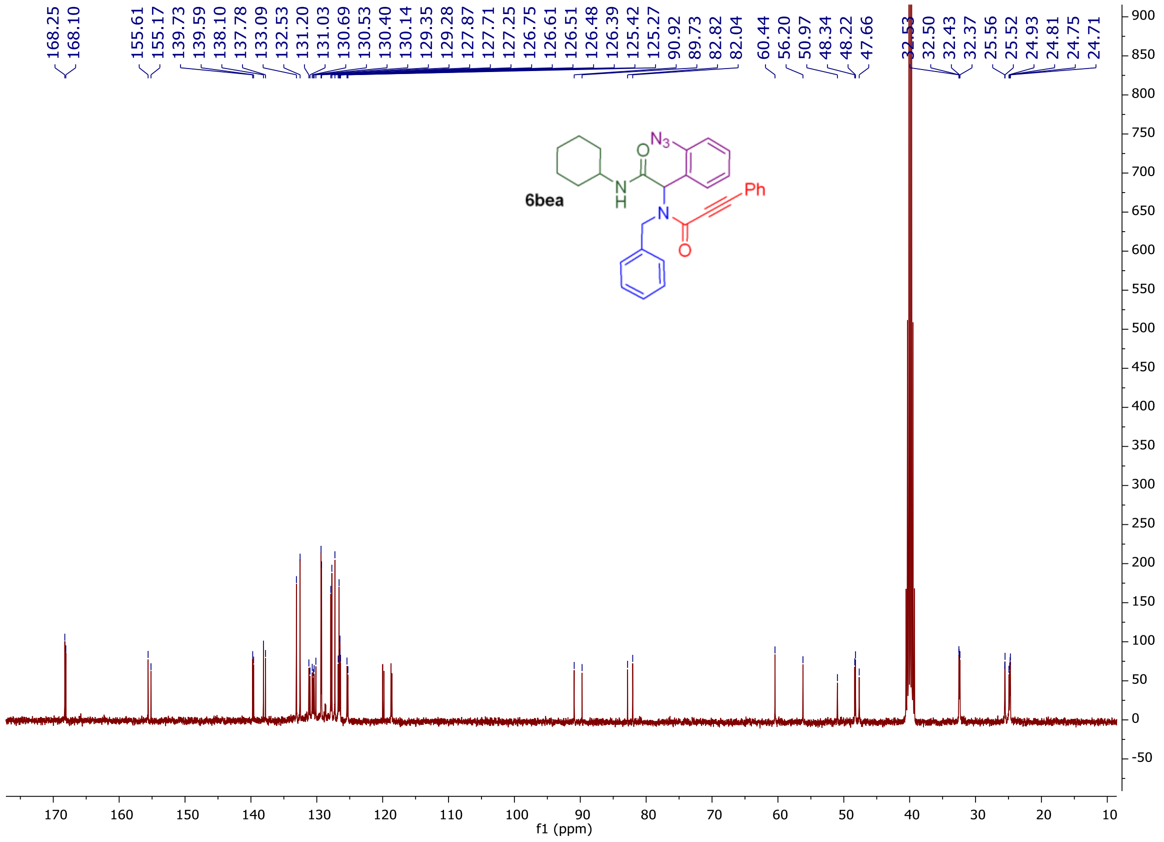

^{13}C NMR spectra of **7aaa**

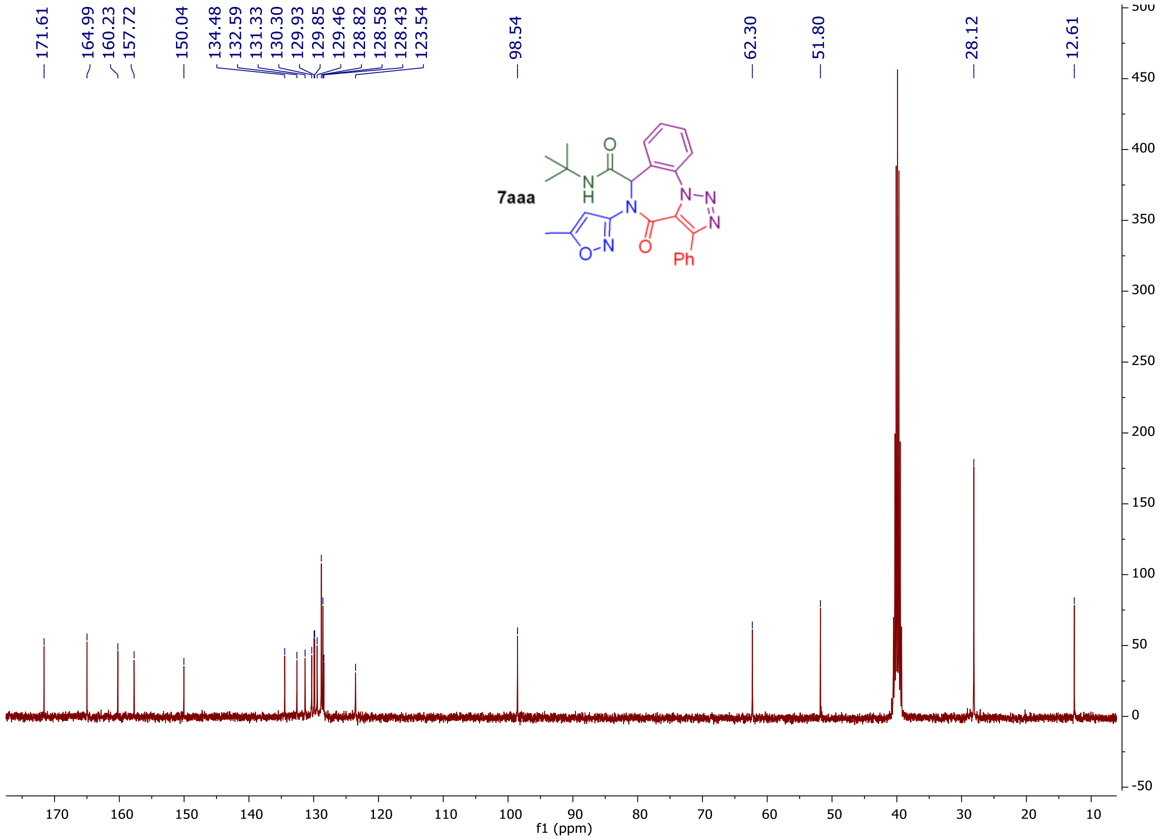

^{13}C NMR spectra of **7aba**

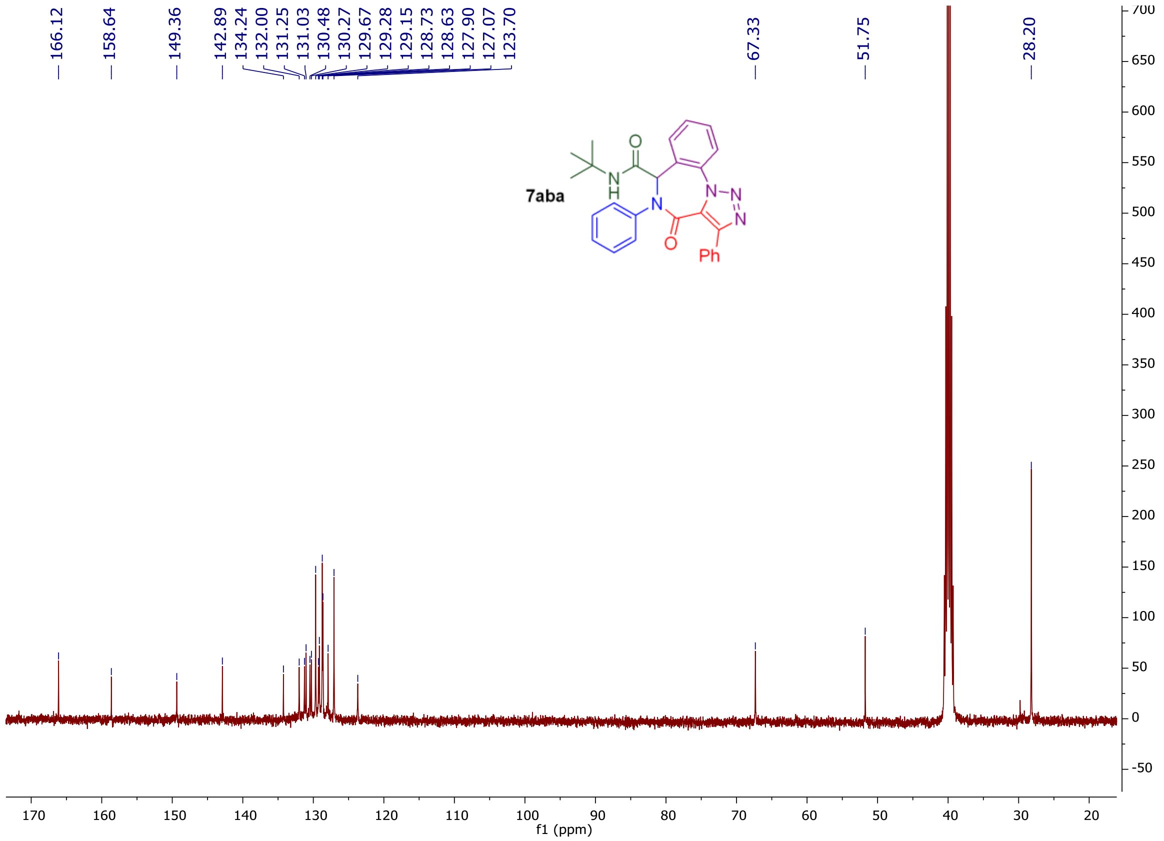

^{13}C NMR spectra of **7aca**

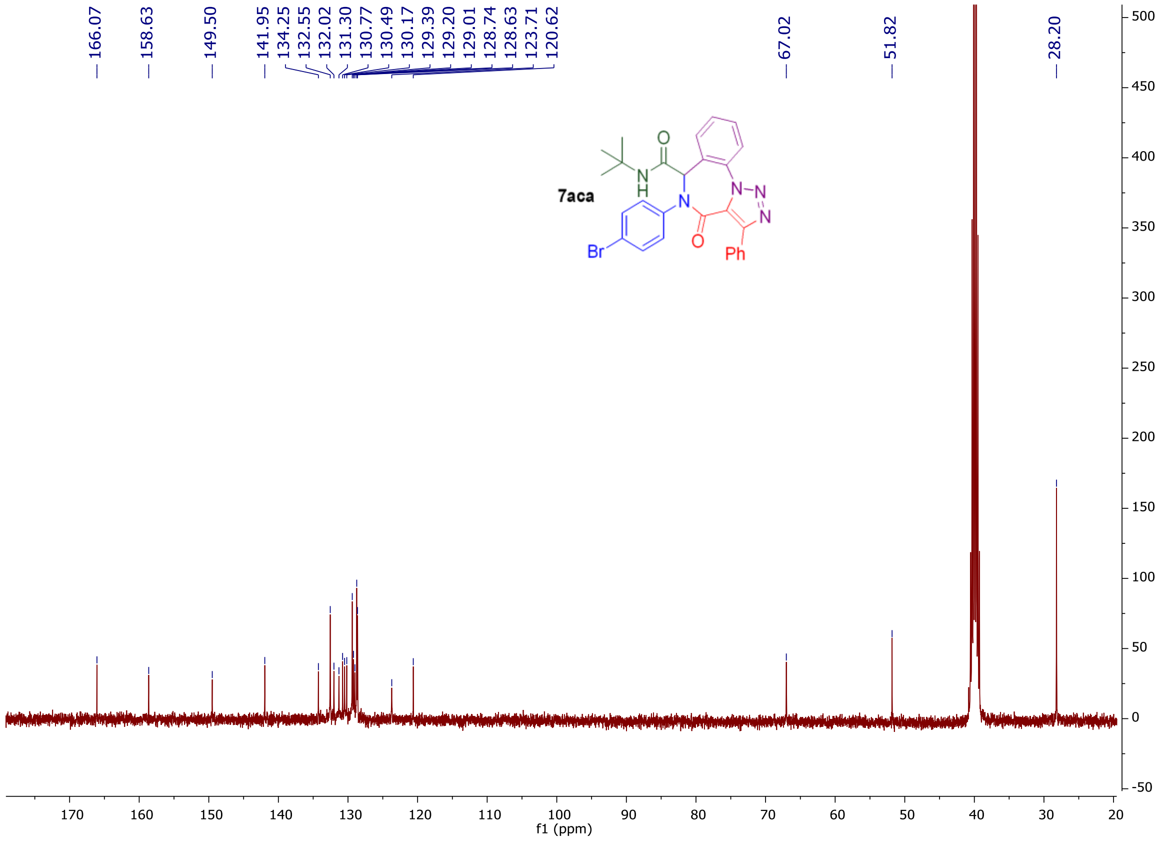

^{13}C NMR spectra of **7ada**

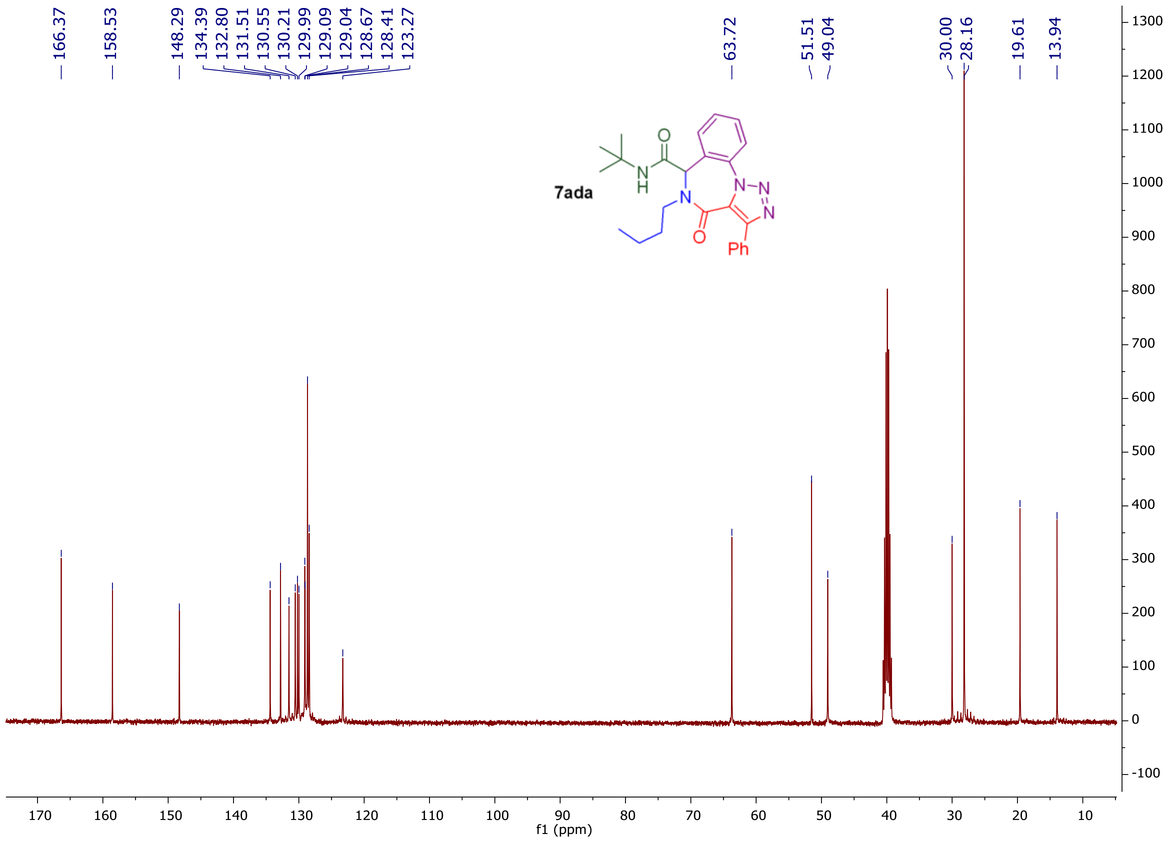

^{13}C NMR spectra of **7aea**

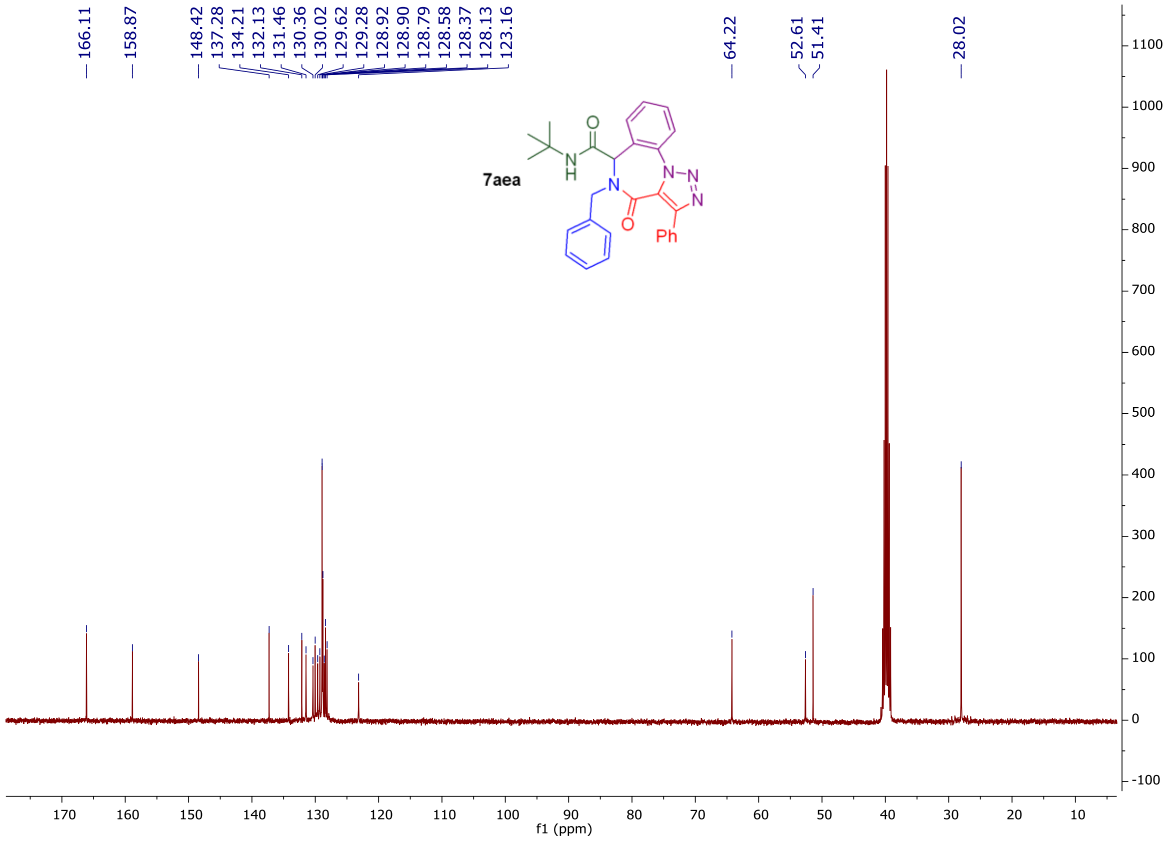

^{13}C NMR spectra of **7aab**

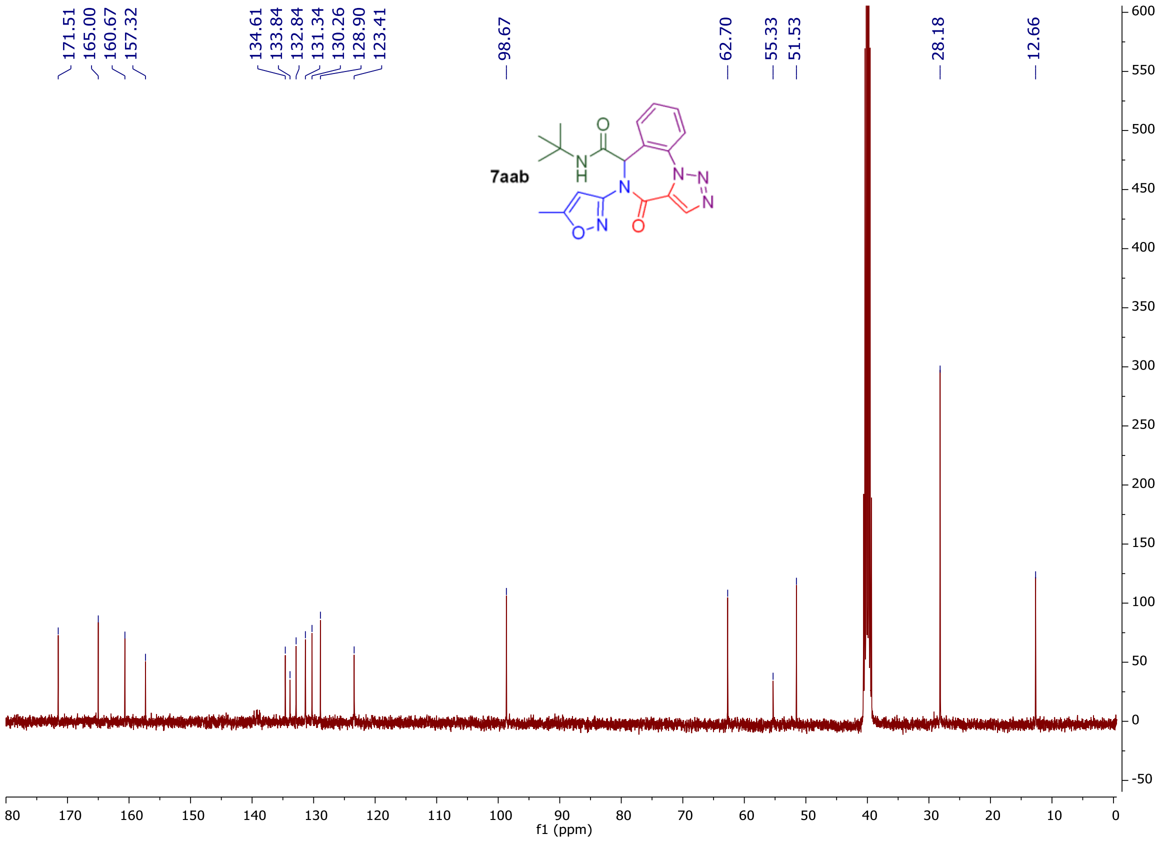

^{13}C NMR spectra of **7abb**

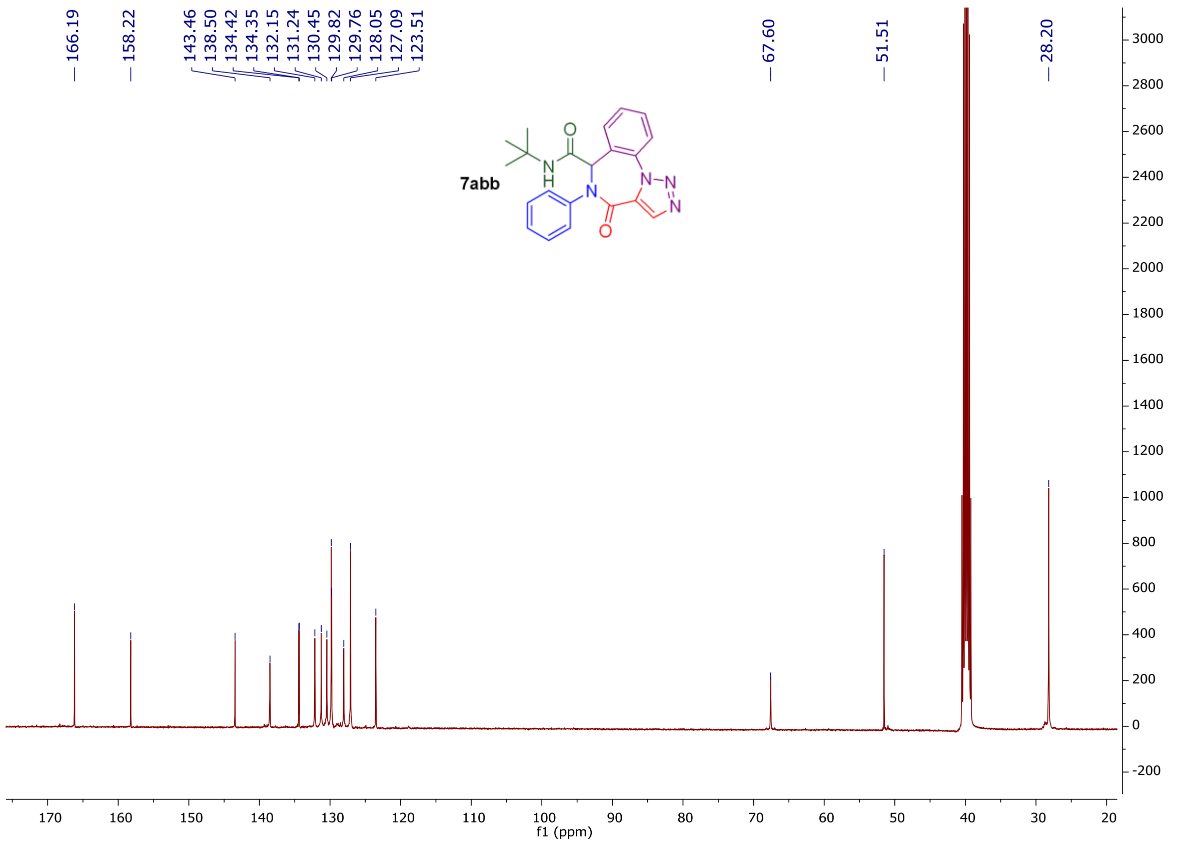

^{13}C NMR spectra of **7aeb**

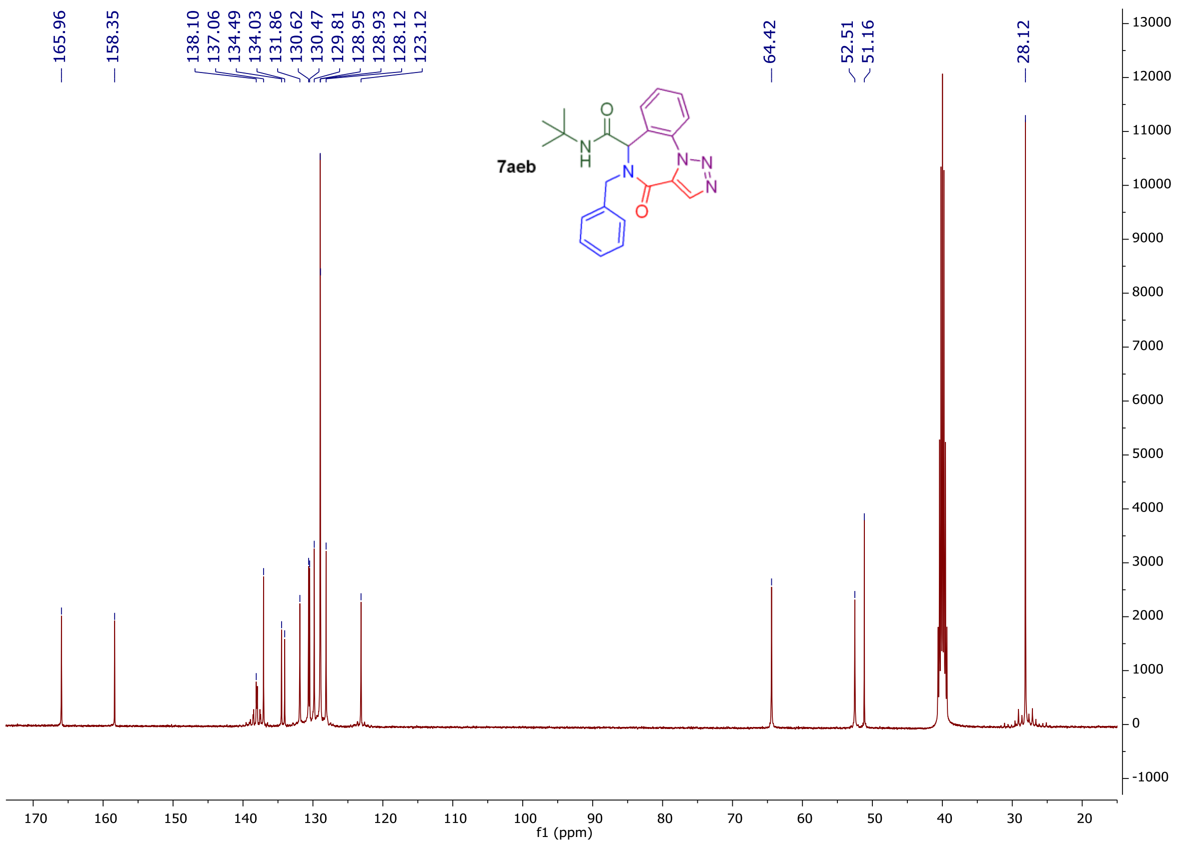

^{13}C NMR spectra of **7baa**

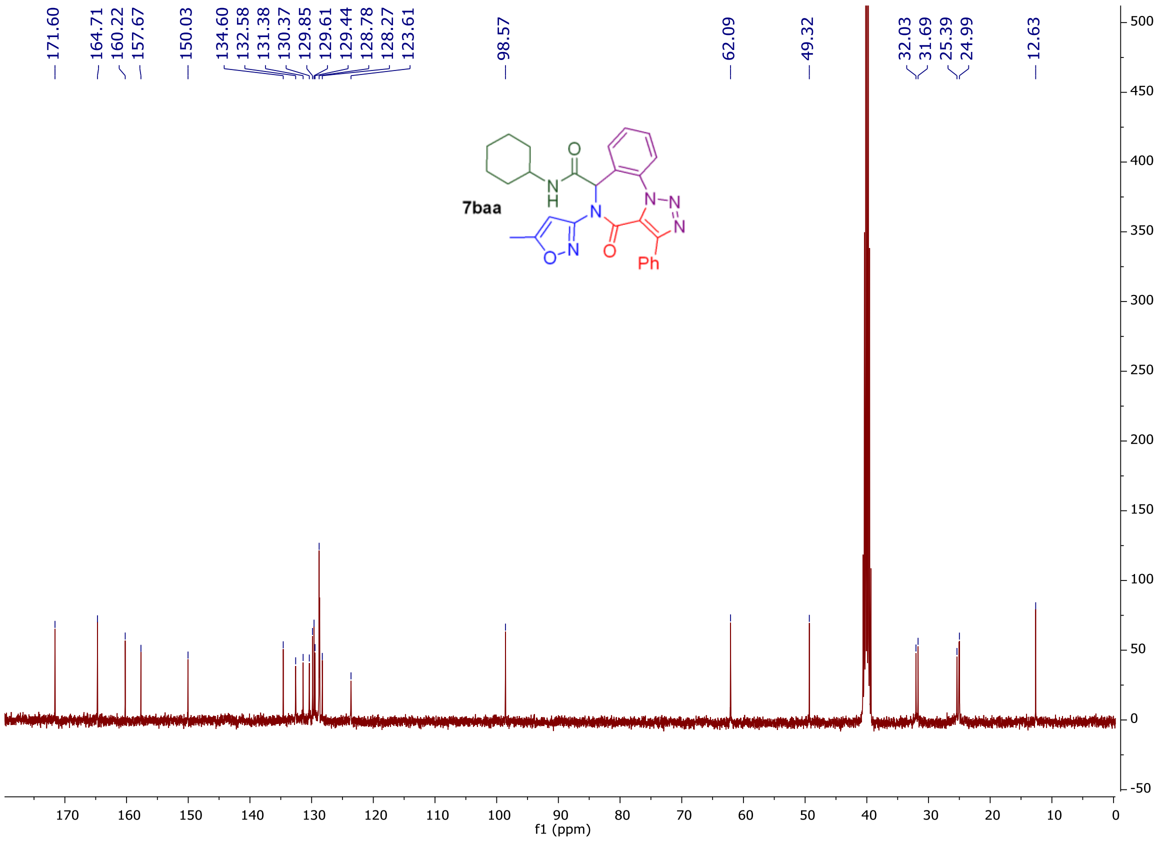

^{13}C NMR spectra of **7bba**

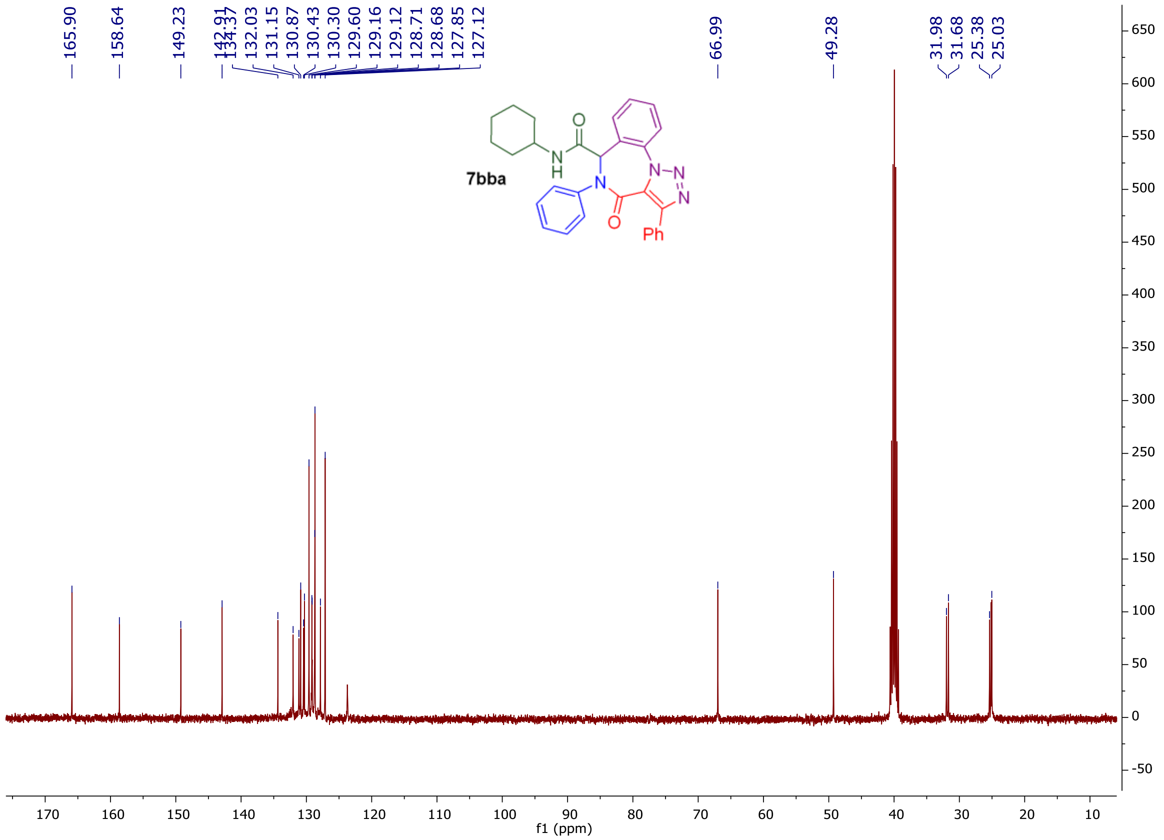

^{13}C NMR spectra of **7bea**

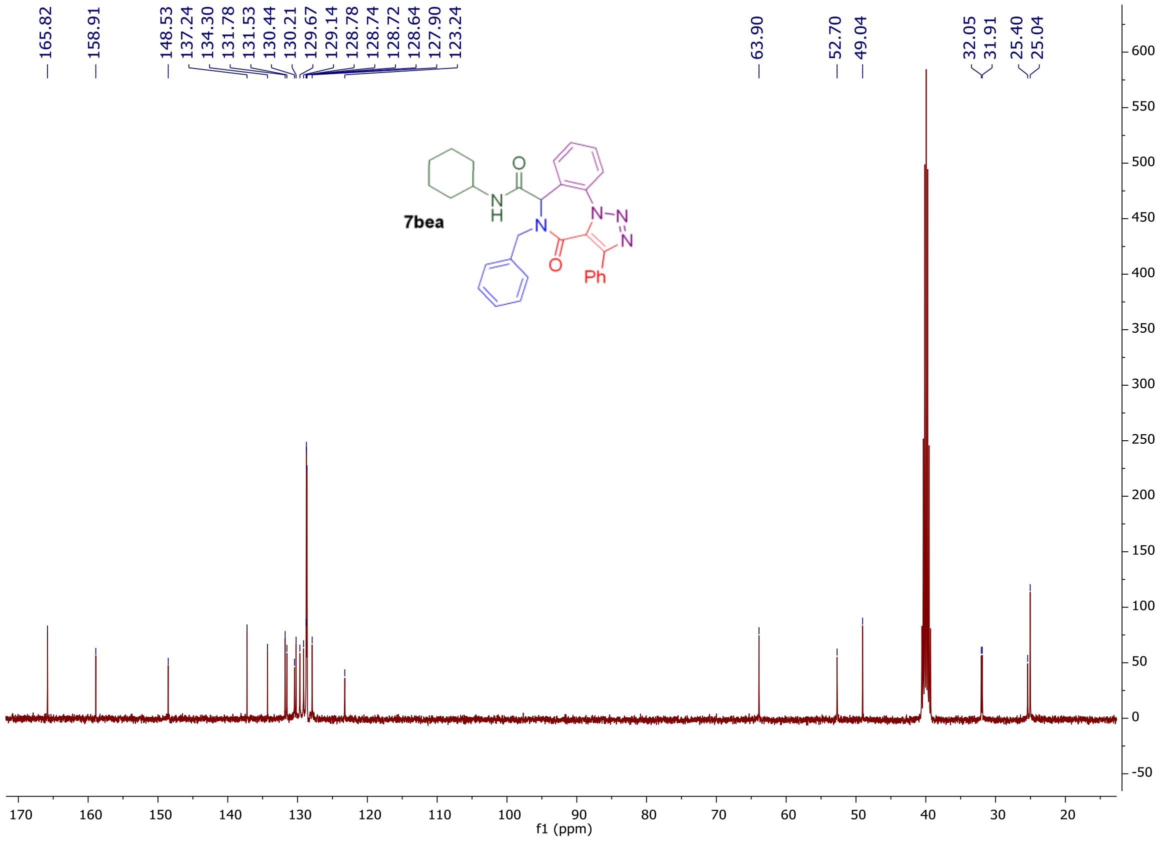

Supplement: File 1 — General synthetic procedures, characteristics of compounds 6 and 7, X-ray experimental data and copies of 1H and 13C NMR spectra. [file Beilstein_J_Org_Chem-17-678-s001.pdf]
